# Spatial distribution of anti-mullerian hormone in females of childbearing age in China under the influence of geographical environmental factors

**DOI:** 10.1186/s12889-023-16431-y

**Published:** 2023-08-18

**Authors:** Wenjie Yang, Miao Ge, Zhujuan Wang, Congxia Wang

**Affiliations:** 1 School of Geographical Sciences and Tourism, Institute of Health Geography, Shaanxi Normal University, Xi’an, 710119 China; 2 Department of Nephrology, Yulin No. 2 Hospital, Yulin, 719000 China; 3 Second Affiliated Hospital, Xi’an Jiaotong University, Xi’an, 710119 China

**Keywords:** Anti-mullerian hormone, Geographical factors, Reproductive Health, Ridge regression, Spatial distribution

## Abstract

The Anti-mullerian hormone (AMH) reference value is an important indicator of ovarian function. The main targets of this were to screen the geographical environmental factors that may influence the distribution of AMH reference values in Chinese females of childbearing age, and to further explore the geographical distribution differences of AMH reference values. We gathered the AMH data of 28,402 healthy Chinese females from 62 cities in China for this study in order to conduct a spearman regression analysis to determine the relationship between the AMH and 30 geography factors. The AMH reference value in different regions was forecasted by using a ridge regression model. The magnitude of influence from the geographical factor on different regions was analysed by geographically weighted regression. Ultimately, We were able to figure out the geographic distribution risk prediction of AMH reference values by utilizing the disjunctive Kriging method. The AMH reference value was significantly correlated with the 16 secondary indexes. The geographical distribution of AMH showed a trend of being higher in Qinghai-Tibet and Southern regions, and lower in the Northwest and Northern regions. This study lays the foundation for future investigations into the mechanism of different influencing factors on the reference value of AMH. It is suggested that such regional variations in AMH reference values be taken into account while diagnosing and treating individuals with reproductive medicine.

**Supplementary Information:**

The online version contains supplementary material available at 10.1186/s12889-023-16431-y.

## Introduction

With the global decline in fertility levels, reproductive health has become an important public health issue in the twenty-first century [1]. Since the implementation of family planning in China, the fertility rate has remained roughly at 1.5 ~ 1.6 in the last decade. However, it dropped to 1.3 for the first time in 2020, which is below the internationally recognized alert level of 1.5 [2]. Fertility decline and aging have become critical challenges for the whole of society. The decline in female fertility is mainly reflected in two aspects: the diminished ovarian reserve function and the occurrence of infertility. The prevalence of infertility in China was about 9% in 1990 [3]and has shown a rapid increase in recent years, from 11.9% in 2007 to 15.5% in 2010 [4]. Meanwhile, the incidence of reduced ovarian reserve function in Childbearing age females is increasing every year [5]. Reproductive health has been an important and emerging area of focus within the natural sciences, social sciences, epidemiology, and obstetrics and gynecology. Studies that address disciplinary boundaries can remove obstacles for researchers to move forward in their full exploration of this issue [6]. Scholars have suggested that the interlinkages between environmental and reproductive debility need greater scrutiny from the perspective of a mixed-methods approach including environmental science, toxicology, and nature and social science [7]. Therefore, from a geographical and environmental perspective, exploring the spatial distribution and influencing factors of female ovarian hormone indicators is of great significance for effectively evaluating ovarian function and fertility potential in females of childbearing age in different regions.

Anti-mullerian hormone (AMH) is an important reference for evaluating ovarian reserve function. It is secreted by the granulosa cells of the antral follicles and the small luminal follicles [8]. Within the normal range, when AMH levels are high, it indicates more oocytes and a longer fertile period, while when AMH levels are low, it indicates poor ovarian function [9]. Compared with other indicators in reproductive medicine, AMH is not affected by menstruation and exogenous steroid hormones, so it is widely used in reproductive medicine, such as assessing female ovarian function, predicting ovarian response, predicting premature ovarian failure, predicting menopause and diagnosing polycystic ovary syndrome. Besides these, it can also be used as a special marker for the diagnosis of ovarian granulosa cell tumors [10, 11].

Some scholars have pointed out in the study that even if the same kit was used for examination, there were still significant differences in AMH reference values of healthy childbearing-age females in different regions. It was also emphasized that the reference value of AMH will vary due to regional and ethnic differences, which should be taken into account in clinical diagnosis [12]. Therefore, to improve the accuracy of clinical diagnosis, since 2016, the regional AMH reference standards of Henan, Xinjiang Karamay, Urumqi, Shaanxi Xi'an, Hubei Huanggang, Guangxi Hechi, Sichuan Chengdu, Guangdong Dongguan, Shanghai and other areas have been established [13–23]. After comparing these reference standards together, we can find that there are differences in the AMH reference values of Chinese females of childbearing age, but throughout the current research, the distribution characteristics of this difference in the country are still unknown.

There are many factors that may lead to differences in AMH values, such as age, BMI, menstrual cycle, and testing methods [24–26]. Apart from these factors, it has been suggested that differences in AMH reference values are related to different geographic environments of people [17, 27]. However, throughout previous studies on the factors affecting AMH, there are currently fewer studies on the effect of geographic environmental factors on AMH. Geographic environmental factors include location, terrain indicators, climate, soil air quality, socioeconomic development level and so on. Health and the living environment are closely intertwined [28]. The influence of geographic environment on the spatial variation in medical reference values, such as vitamin D, activated partial thromboplastin time, etc., has been demonstrated in many studies [29–31].

Consequently, this study built an index system to filter the factors which influence serum AMH reference value in Chinese females of reproductive age from the perspective of the geographical environment. By building a model, AMH in Chinese females of childbearing age in various locations was estimated. To investigate the distribution of the serum AMH reference value, geostatistical analysis was utilized. Finally, it investigated how regional environmental factors affected the distribution of the AMH.

## Materials and methods

### Data sources

**Fig. 1:**
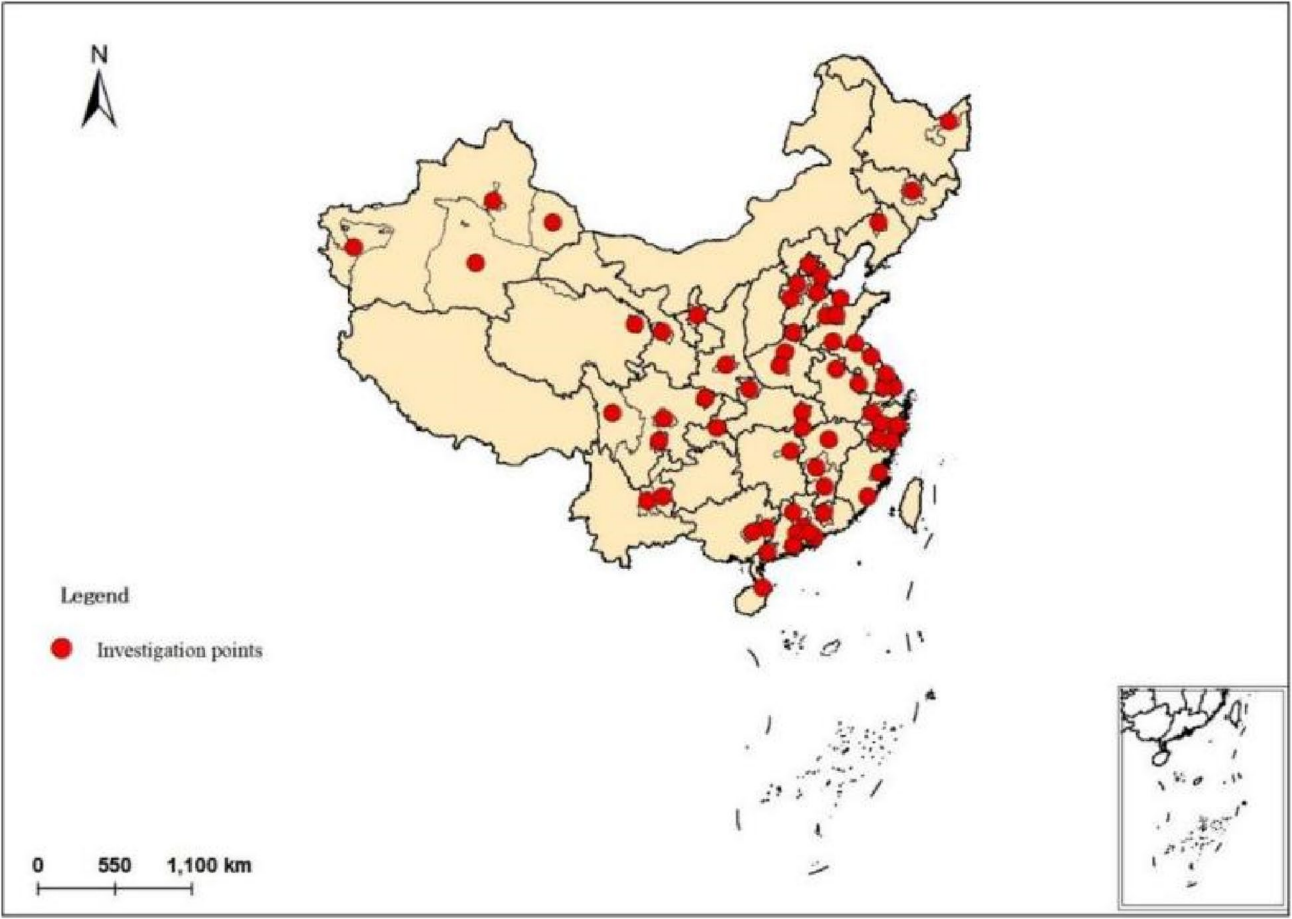
The distribution of investigation points

The keywords "AMH" were searched in a database of journals. Overall, 28,402 serum AMH values were obtained from healthy Chinese females aged 25–35 years (Fig. 1). The distribution of the sample data coincides with the distribution of population density in China, with more data from the east than from the west. In addition, some regions are sparsely populated and lack of medical resources, resulting in smaller sample data for these locations (Table 1). All these data were measured by Enzyme-linked immunosorbent assay (ELISA). The unit was ng/ml. Age is one of the most important factors affecting AMH values in females, and the inflection point for AMH decline is after 35 years old [32]. Therefore, to control for individual age differences, a more stable age group was selected for the study and all the study subjects in this paper were selected between the ages of 25–35. All data were experimental data obtained from published articles, which are displayed in the Appendix.

**Table 1:** Distribution of reference value of AMH

Provinces or Municipalities	Number of cities and counties included	Sample sizes (people)	Provinces or Municipalities	Number of cities and counties included	Sample sizes (people)
Anhui	1	40	Jiangxi	3	469
Beijing	1	396	Liaoning	1	2400
Fujian	2	1040	Ningxia	1	279
Gansu	1	100	Tianjin	1	110
Guangdong	7	3889	Qinghai	1	50
Guangxi	2	137	Shandong	4	506
Jilin	1	46	Shaanxi	1	709
Hainan	2	100	Sichuan	4	1210
Hebei	3	388	Xinjiang	4	2466
Henan	3	5461	Yunnan	2	188
Heilongjiang	1	30	Zhejiang	5	612
Hubei	3	4219	Chongqing	1	66
Hunan	1	100	Jiangsu	4	1161
Shanghai	1	1370	Shenzhen	1	860

The geographic indicators we chose were spatial location, terrain indicators, climate, air quality, soil qualities and social economy level (Table 2). We separated them into 30 sub-indices. Data on AQI, CO, NO2, SO2, PM2.5, and PM10 were gathered from 1496 national ambient air quality monitoring stations in China. Urban regions are where most air quality monitoring stations are found. Six contaminants' hourly data were included in the data, along with a daily 24-h moving average. The meteorological and atmospheric pollutant point data covering the study area were processed using kriging interpolation and zonal statistics based on AMH-level data at the municipal level to ensure a matching between the data accordingly, which can then be used for modeling.

**Table 2:** The Geographical environmental indicators

Type	The name and unit of the indicator	Sources
Location	Longitude (°)	The National Bureau of Surveying and Mapping. (http://www.nasg.gov.cn/)
	Latitude (°)	
Terrain	Altitude (m)	
Climate	Annual sunshine duration (h)	The China Meteorological Science Data Sharing Service Network. (http://cdc.cma.gov.cn)
	Annual mean temperature (°C)	
	Annual mean relative humidity(%)	
	Annual recipitation (mm)	
	Annual temperature range (°C)	
Soil	Percentage of gravel in topsoil (% wt)	
	Topsoil silt percentage (% wt)	
	Percentage of silt in topsoil (% wt)	
	Calcium sulfate content of topsoil (%)	
	The alkalinity of topsoil (cmol/kg)	
	The Salinity of topsoil (dS/m)	
	Reference bulk density of topsoil (kg/dm^3^)	
	Gravel content of topsoil (% vol)	
	Organic matter content of topsoil (% wt)	
	pH value of topsoil	
	The cation exchange capacity of topsoil (cmol/kg)	
	Base saturation of topsoil (%)	
	Total capacity of topsoil (cmol/kg)	
	Calcium carbonate content of topsoil (%)	
Air Quality	AQI	Gathered from 1496 national ambient air quality monitoring stations in China
	PM_2.5_(μg/m^3^)	
	PM_10_(μg/m^3^)	
	SO_2_(μg/m^3^)	
	CO(μg/m^3^)	
	NO_2_(mg/m^3^)	
Social economy	Population density(People/km^2^)	Gathered from the statistical yearbooks of 34 provincial administrations in China
	Real GDP per capita	

## Methods

### Spatial autocorrelation analysis

Spatial autocorrelation analysis is an important component of spatial statistics and an effective method for understanding spatial patterns. In addition to exposing the regional structural patterns of spatial variables, this efficient spatial statistical method can also determine whether the attribute values of an element are related to those of its nearby spatial points [33]. There, it is applied to investigate whether there is a correlation between AMH data of its neighboring spatial points. The judgment is based on the value of its output Moran's I and Z scores. The following equation is used to calculate Moran's *I*: (1).

1 $$I=\frac{n{\displaystyle\sum\limits_{i=1}^n}{\displaystyle\sum\limits_{j=1}^n}w_{ij}\left(y_i-\overline y\right)\left(y_j-\overline y\right)}{\left({\displaystyle\sum\limits_{i=1}^n}{\displaystyle\sum\limits_{j=1}^n}w_{ij}\right){\displaystyle\sum\limits_{i=1}^n}\left(y_i-\overline y\right)^2}$$

In the formula,* n* denotes the total amount of samples for a given variable. The observations of the variables in regions *i, j* respectively are denoted as *y*_*i*_*,y*_*j*_*. W*_*ij*_ denotes the elements of the spatial weighting matrix.

The* Z*-score is calculated by using the following formula (2).

2 $$Z=\frac{I-E\left(I\right)}{\sqrt{Var\left(I\right)}}$$

#### Local spatial autocorrelation

Local spatial correlation index is a method to investigate the of clustering or abnormalities of values within a local area [34]. It can help reveal the degree of spatial autocorrelation between the AMH reference values of each study unit and its neighboring units. It is calculated by using the following formula (3).

3 $$I_{\text{i}}=\frac{n\left(x_j-\overline x\right){\displaystyle\sum\limits_{i=1}^n}w_{ij}\left(x_i-\overline x\right)}{{\displaystyle\sum\limits_{j=1}^n}\left(x_j-\overline x\right)^2}$$

*I*_*i*_ represents the Local Moran′s I index, and the rest of the symbols have the same meaning as above.

#### Correlation analysis

Correlation analysis is a convenient and effective method of measuring the relationship between several groups of quantitative data. It can examine the correlation between variables as well as the strength of the correlation. Pearson's correlation coefficient, Spearman's rank correlation coefficient, and Kendall's correlation coefficient are the three most popular types of correlation coefficients. The most often utilized of these is the Pearson correlation coefficient, while the Kendall correlation coefficient is used to assess data consistency, such as judge scoring, and the Spearman correlation coefficient is used when the data does not satisfy normality [35]. Here, the Spearman rank correlation coefficient was chosen, and the coefficient was derived by the formula below.

4 $$r=1-\frac{6{\displaystyle\sum\limits_{i=1}^n}d_i^2}{n\left(n^2-1\right)}$$

The grade difference is d_*i*_, and the sample size is *n*.

#### Ridge regression analysis

Ridge regression analysis is a more accurate version of least squares that is more in accordance with the data [36]. Herein, it was used to build a predictive mode. The reference value for AMH served as the dependent variable, with the pertinent geographic factors acting as independent variables.

The Wilcoxon signed-rank test is a refinement of the signed test method in non-parametric statistics. It not only makes use of the positive or negative difference between the observed value and the central position of the original hypothesis, but also makes use of information about the magnitude of the value difference. This method of testing has three advantages. Firstly, although it is a simple non-parametric method, it embodies the basic idea of rank. Secondly, it takes the rank of the absolute value of the difference between the observed value and the central position of the null hypothesis and adds them separately according to different signs as its test statistic. Thirdly, it is applicable to pairwise comparisons in t-tests, but does not require the difference between pairs of data with a normal distribution, only a symmetric distribution [37].

#### Geographically weighted regression (GWR) model

The geographically weighted regression model is a kind of local regression model. Compared with other traditional global regression models, its advantage is that local regression coefficients can be obtained for different geographical units [38]. Here, it was used to find out the intensity of the impact of the same environmental factor on different geographical units. The GWR tool in ArcGIS 10.2 software was used to model the relationship between environmental factors and AMH reference values. (https://www.esri.com/en-us/home).

## Results

### AMH spatial distribution

#### Spatial autocorrelation analysis

**Fig. 2:**
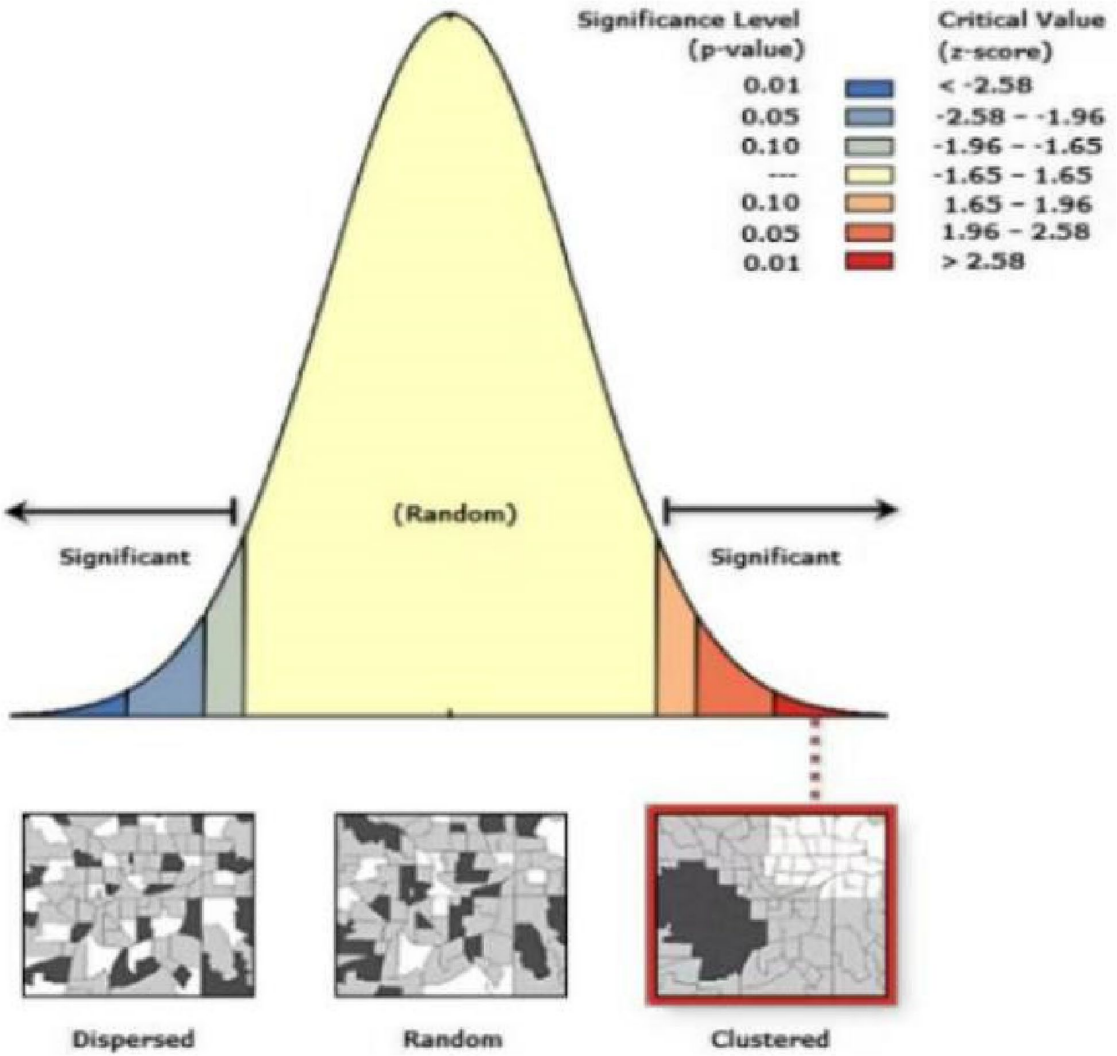
Result of spatial autocorrelation analysis

The Moran index (Moran *I)* was 0.949 (> 0). The global autocorrelation index *Z* was 8.296 (> 2.580) and the probability value *P* was 0.000 (Fig. 2). The findings of the spatial autocorrelation analysis revealed a correlation between the serum AMH reference value and spatial locations. There were regional variations in serum AMH.

#### local autocorrelation analysis

**Fig. 3:**
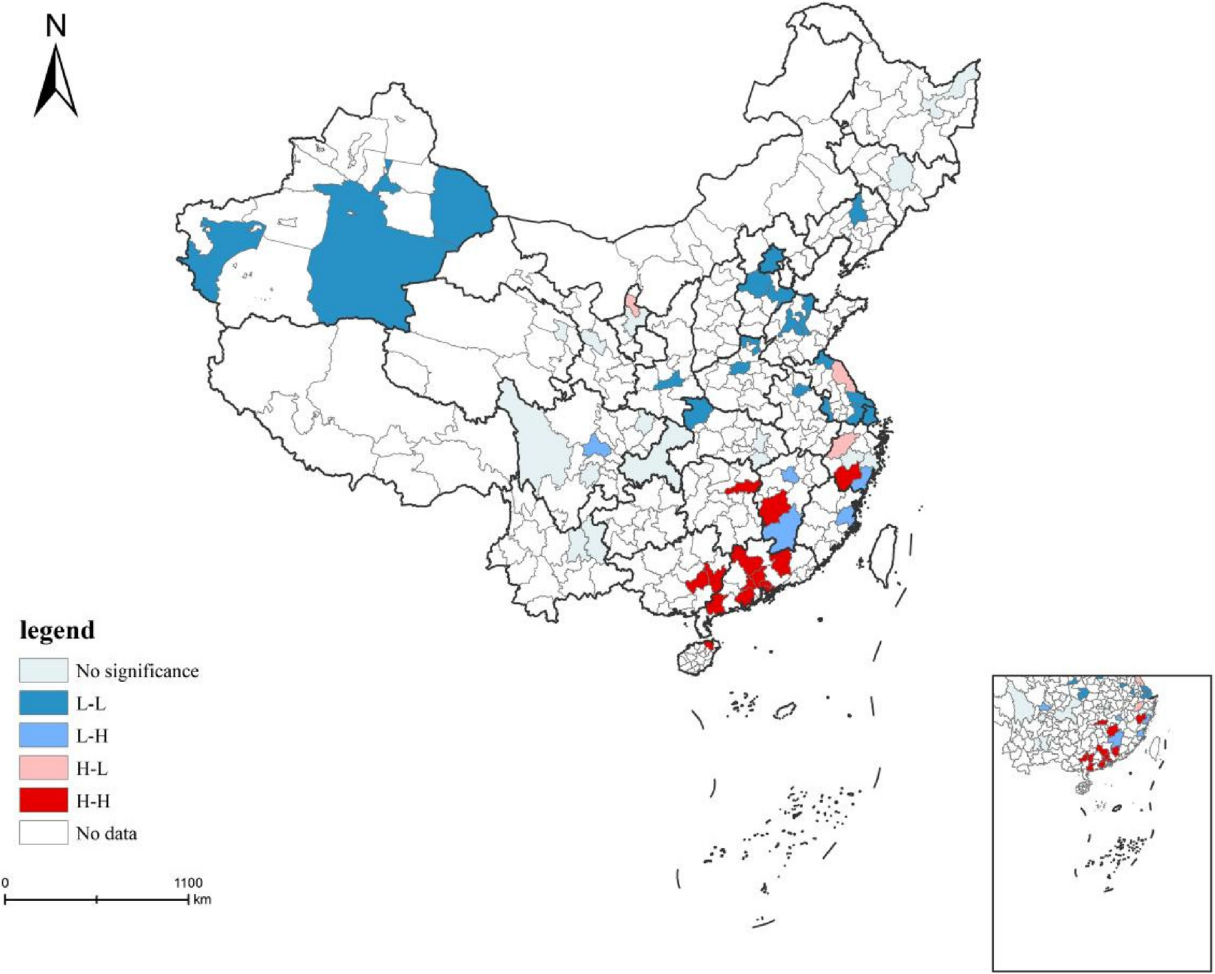
LISA plots of AMH reference values

To further explore the local spatial pattern of AMH reference values, the Local Indicators of Spatial Association (LISA) plot was drawn by using GeoDa software (Fig. 3). It can be discovered that the clustering distribution of the AMH reference value differs spatially from north to south. The H–H regions are mainly located in southern China, including Heyuan, Shenzhen, Jiangmen, Maoming, Qingyuan, Haikou, Wuzhou, etc. L-L regions are mainly located in northern China, including Urumqi, Shenyang, Beijing, Shijiazhuang, and Shanghai. L–H and H–L regions are scattered in southern China. L–H regions include Chengdu, Xi'an, Nanchang, Jiangxi, Ganzhou, Fuzhou, Fujian, Wenzhou, Zhejiang. H–L regions include Hangzhou, Yancheng, and Yinchuan.

### Correlation analysis

The geographic factors and the AMH reference value were discovered by spearman correlation analysis. The relationship between geographic characteristics and the AMH reference value was evaluated using the correlation coefficient (*r*) and significance coefficient (*P*). Through the values, it can be clearly found that there are 16 geographical factors that have a correlation with serum AMH reference value. (Table. 3).

**Table 3:** Correlation coefficient between AMH and geographical factors

Symbol	Geographic factors	*r* value	*P* value
X_1_	Latitude (°)	-0.332^b^	0.000
X_2_	Annual mean temperature (℃)	0.249^b^	0.001
X_3_	Annual mean relative humidity (%)	0.180^a^	0.021
X_4_	Annual precipitation (mm)	0.307^b^	0.000
X_5_	Annual temperature range (℃)	-0.317^b^	0.000
X_6_	AQI	-0.418^b^	0.000
X_7_	PM_2.5_	-0.433^b^	0.000
X_8_	PM_10_	-0.406^b^	0.000
X_9_	SO_2_	-0.182^a^	0.014
X_10_	CO	-0.320^b^	0.000
X_11_	NO_2_	-0.334^b^	0.000
X_12_	Total capacity of topsoil	-0147^a^	0.027
X_13_	Percentage of silt in topsoil	-0.160^a^	0.031
X_14_	Calcium sulfate content of topsoil	-0.185^a^	0.013
X_15_	Percentage of gravel in topsoil	-0.211**	0.004
X_16_	The alkalinity of topsoil	-0.157^a^	0.034

^a^represents correlation, ^b^represents the significant correlation

### Model establishment

#### Ridge regression analysis

**Fig. 4:**
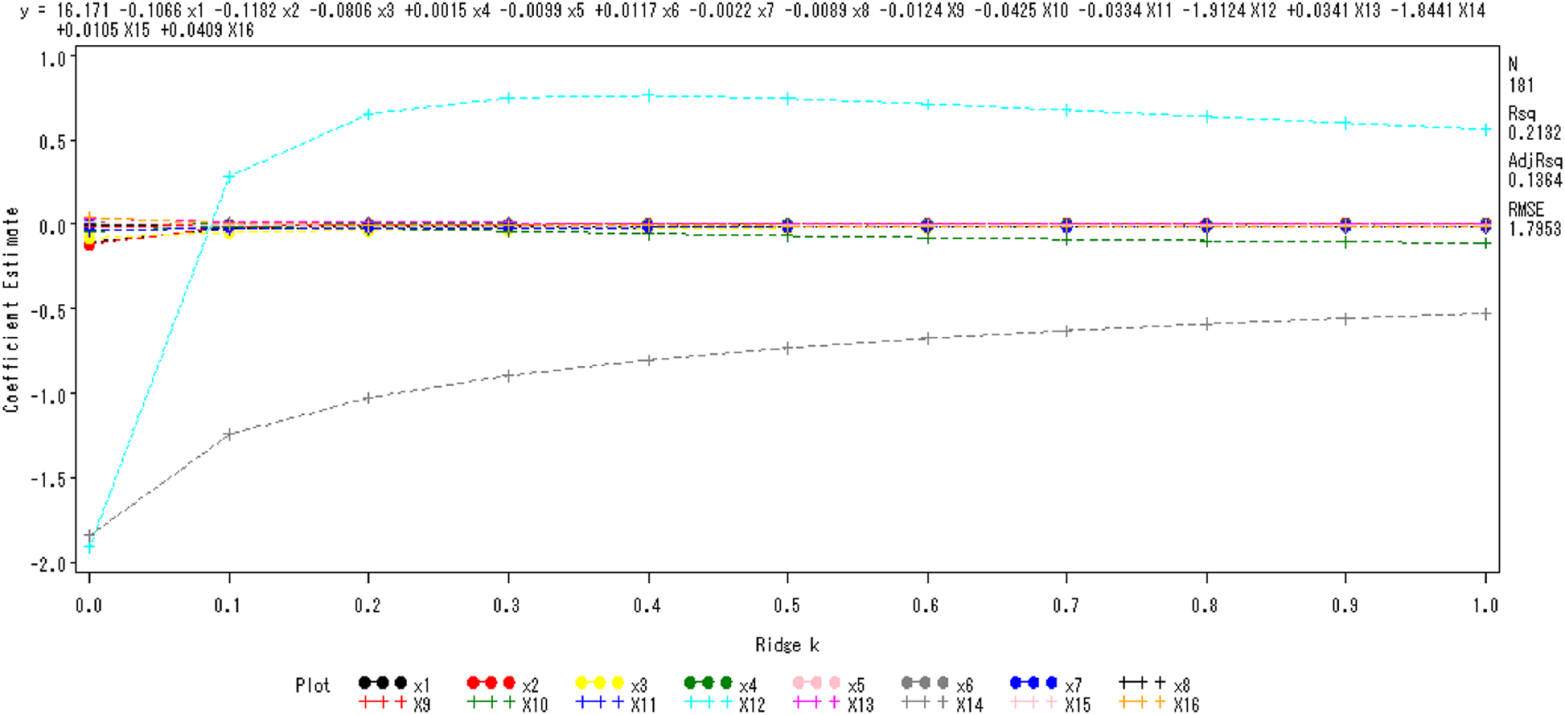
Ridge trace map of the reference value of serum AMH in healthy females of childbearing age

A ridge regression model was created using the aforementioned 16 geographic characteristics as independent variables and the reference value of serum AMH as the dependent variable (Fig. 4).

The ridge trace parameter is shown by the horizontal axis, and the regression coefficient for each factor is represented by the vertical axis. The trend of each factor's ridge trace is rather constant when the ridge parameter K = 0.3, and the following regression equation is thus produced.

5 $$\widehat Y = 7.6849-0.0144X_{1}-0.0006X_{2}-0.02733X_{3}+0.0003X_{4}-0.01292X_{5}-0.0072X_{6}-0.0091X_{7}-0.0042X_{8}-0.00357X_{9}-0.04021X_{10}-0.02080X_{11}-0.75010X_{12}+0.00963X_{13}-0.89583X_{14}-0.00499X_{15}-0.00661\pm 1.82035$$

*Ŷ* is the serum AMH reference value (ng/ml), *1.82035* is the remaining standard deviation.

MSE, MAE, RMSE(E), Standard Deviation (SD) and R^2^ are often used as important metrics to evaluate the quality of the model (Table 4). R^2^ takes values in the range of [0, 1]. The larger the R-Squared is, the better the model fit is. Here the R^2^ of this model was 0.482.

**Table 4:** Model quantitative evaluation

Metrics	MSE(Mean Square Error)	MAE(Mean Absolute Error)	RMSE(E)	Standard Deviation (SD)	R^2^
Value	2.470	1.197	1.571	0.745	0.482

**Fig. 5:**
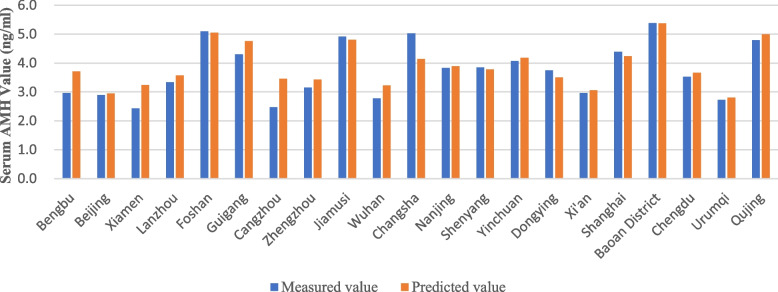
Comparison of measured and predicted values of AMH

The results of wilcoxon signed rank test showed that* P* was *0.528* (> 0.05). It indicated that there was no significant difference between the predicted value & measured value. A table was made to show Comparison of serum AMH measured and predicted reference values in 21 cities across the country (Fig. 5).

#### Geographically weighted regression (GWR) model

Although correlation analysis can find out probable factors that may be associated with AMH, it is unable to estimate the magnitude from each factor's impact on AMH in various geographic locations.Therefore, in order to estimate the impact of a single geographic factor on various locations, the GWR model has been used here. 10 geographic factors's variance-inflated factor (VIF) values exceeded 7.5, which indicated that there were co-linearities between these factors.To avoid the results from being distorted by factor co-linearity, these factors need to be removed.The left 6 factors were analysed by geographically weighted regression.

**Fig. 6:**
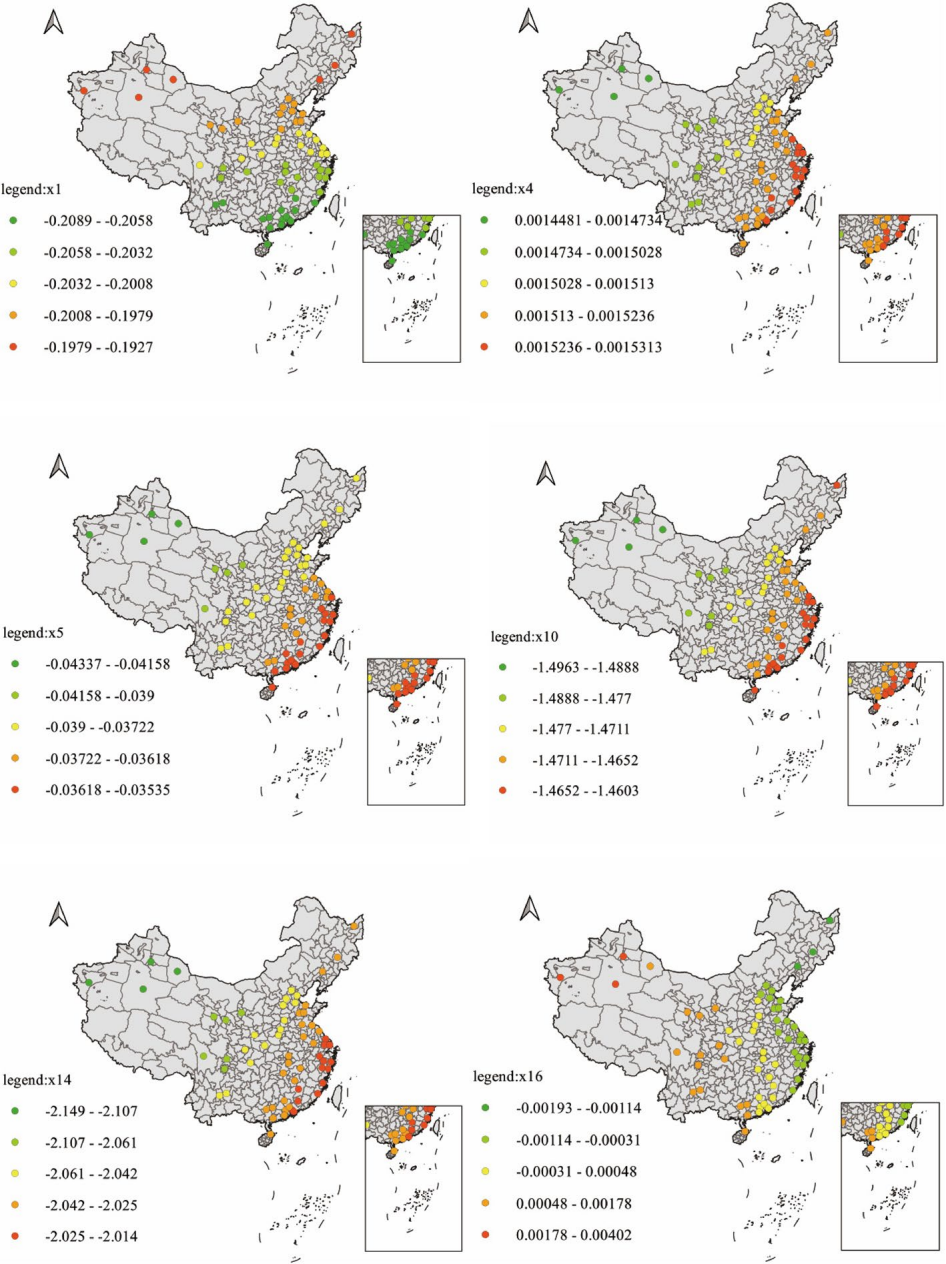
GWR results of AMH reference value and environmental factors

R^2^ for the GWR ftting was 0.3817. There is an overall negative correlation between latitude and AMH, and this negative correlation gradually increases from south to north. Both Annual temperature range (°C) and Annual precipitation (mm) were positively correlated with AMH reference values, and there were sea-land differences in the effects of these two factors. Both have a stronger influence on AMH reference values for females in the southeast coastal region, but a lower influence on AMH reference values for females in the northwest. Northwest China is inland, far from the ocean, and the surrounding mountains block the arrival of oceanic air currents, exhibiting typical temperate continental climate characteristics. The climate is characterized by low precipitation, dryness and a large annual difference in temperature, whereas the coastal areas of southeast China have a subtropical monsoon climate with abundant rainfall and distinct wet and dry seasons. This leads to the conclusion that arid regions with low precipitation and large annual differences in temperature are more influenced by the Annual precipitation (mm) factor than humid regions with sufficient precipitation and relatively low annual differences in temperature. The air quality factor CO has an overall negative correlation with the AMH reference value, with a greater effect in eastern regions than in western China. Yupeng et al.found that CO pollution is more serious in eastern regions, two to three times more than in western regions, mainly concentrated in the Yangtze River Delta, Pearl River Delta and Northeast China. Therefore, in these regions AMH reference values are more influenced by CO concentration [39].The increase in calcium sulfate content of topsoil had an inhibitory effect on AMH reference values, with a greater effect in the southeast and a lesser inhibitory effect in the northwest. The alkalinity of topsoil showed a negative correlation in general. An increase in the alkalinity of topsoil also had an inhibitory effect on the increase in AMH reference values, but the local regression coefficients showed variability in different regions of China. In eastern China, the effect of soil alkalinity on AMH reference values showed a more pronounced negative correlation, and the strength of the negative correlation tended to diminish from coastal to inland. In the western part of China, the effect of soil alkalinity on AMH shows a more obvious positive correlation, and the strength of the positive correlation tends to decrease in steps from the Himalaya to central China. It is therefore concluded that in areas with high soil alkalinity, the relationship between soil alkalinity and the AMH reference value shows a positive correlation, while in areas with low soil alkalinity, the relationship between soil alkalinity and the AMH reference value shows a negative correlation (Fig. 6). GWR coefficient estimates results of AMH and influencing factors is shown in Table 5.

**Table 5:** Summary of GWR coefficient estimates

Classifcation	Min	1st Qu	Median	3rd Qu	max
X_1_	-0.2089	-0.2058	-0.2032	-0.2008	-0.1927
X_4_	0.00144	0.00147	0.00150	0.00151	0.00153
X_5_	-0.0433	-0.0380	-0.0372	-0.0361	-0.0353
X_10_	-1.4963	-1.4730	-1.4680	-1.4640	-1.4603
X_14_	0.01784	0.01815	0.01883	0.01918	0.01992
X_16_	-0.00193	-0.00114	-0.00031	0.00178	0.00402

### Spatial distribution risk prediction

**Fig. 7:**
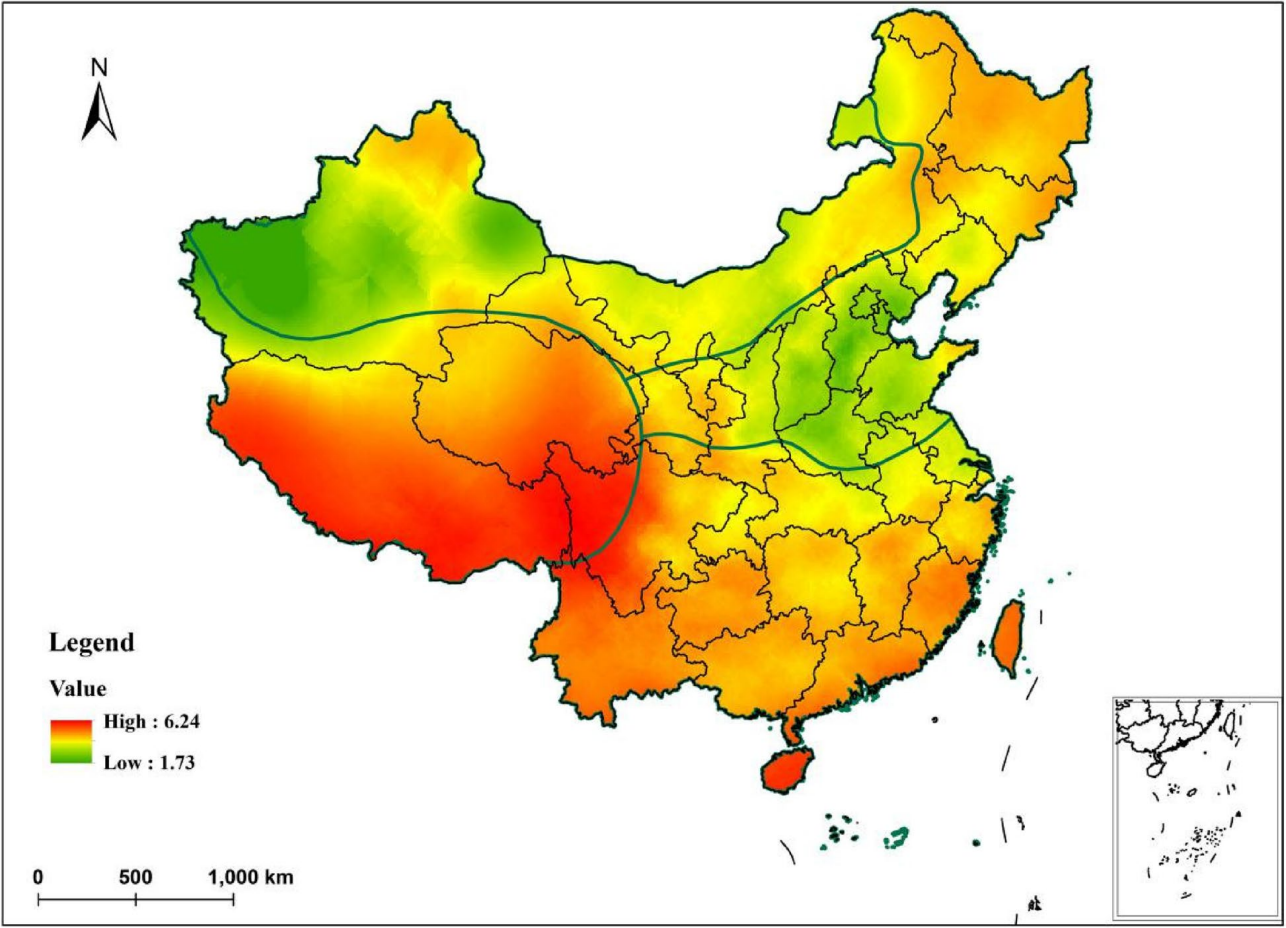
Prediction of Geographic distribution of serum AMH reference values in healthy females of childbearing age in China

Compared with the geographically weighted regression model, the ridge regression model has a higher R^2^ and provides a better fit for this data set. Therefore, we used ridge regression analysis to calculate predicted AMH reference values for 2322 points in China and represented these predictions on maps by using kriging interpolation. Using spatial distribution maps to further characterize the distribution of AMH reference values. The AMH reference values are high in the red-leaning regions and low in the green-leaning regions, and similar color tones indicate small differences in reference values (Fig. 7). This distribution is generally consistent with the results of local autocorrelation aggregation of sample sites.

The geographical distribution of AMH reference values is generally characterised by high values in the west and low values in the east. Based on the characteristics of geographical location, physical geography and human geography, China can be divided into four major geographical regions, Qinghai-Tibet region, Southern region, Northern region and Northwest region. The high values are mainly concentrated in the Qinghai-Tibetan region and the southern region, while the low values are mainly distributed in the northern region and the northwestern region of China. AMH reference values are high in Qinghai-Tibet, Yunnan-Guizhou and other coastal cities in southwest China, but low in Shandong, Shanxi, Hebei and northern Shaanxi and western Xinjiang. Furthermore, taking the Qinling-Huaihe line as the dividing line, the AMH is higher in the north than in the south.

## Discussion

A general decline in fertility is currently taking place globally. According to a study in the Lancet, with widespread fertility decline, 183 of the world's 195 countries and territories will have total fertility below replacement level by 2100 [40]. Among them, China's total fertility rate has already started to enter the ranks of the ultra-low fertility level, which indicates that the country is facing many challenges arising from the serious risk of low fertility [41]. The ovaries and uterus play a very important role in the reproduction of human beings. Female fertility is mainly assessed based on ovarian reserve function. Once ovarian reserve function decreases, the quantity and quality of ova produced by the ovaries will decrease, as will the ability to secrete sex hormones, which ultimately reduces fecundity [42]. Therefore, it is very valuable to investigate the spatial distribution differences of female sex hormone reference values and the influencing factors which can effectively help assess their ovarian function and fertility potential.

In this study, we selected AMH, a sensitive index of ovarian reserve function, as the subject of study. From a geographical perspective, we investigated the effects of geographic environmental factors (including terrain indicators, climate, soil, air quality and social economic level) on AMH reference values. Furthermore, we used ridge regression to model the impact factors across China and used geographically weighted regression to quantify differences in the impact of the same geographic factor across different regions of China. Finally, we used kriging interpolation to create a spatial distribution map of AMH reference values in Chinese females of childbearing age. It can be find that there were regional differences in AMH reference values, which were lower in the north and northwest of China, but higher in southern regions. This distribution was confirmed by the comparison of already established AMH regional reference value standards in cities or provinces such as Henan, Xinjiang Karamay, Urumqi, Shaanxi Xi'an, Hubei Huanggang, Guangxi Hechi, Sichuan Chengdu, Guangdong Dongguan, Shanghai [13–23].

Based on the results of the correlation analysis and geographically weighted regression analysis, we found that this distribution may be caused by several factors. Northern and northwestern regions, with higher latitude and Annual temperature range (℃), these factors can increase the red blood cell content and blood viscosity in the body, resulting in a prolonged adverse environment for the ovaries, thus affecting the AMH reference value [15]. Compared with the northern region, the southern region had a higher annual mean temperature (°C), annual mean relative humidity (%), and annual precipitation (mm), while the female AMH reference value was also higher. At present, there are no human biological experiments to clarify their association, but in experiments on animals by Wan Tao et al. It has been found that AMH levels are higher in a warm and rainy environment with suitable humidity, and lower in winter. It is hypothesized that prolonged exposure of the ovaries to this comfortable environment leads to a positive effect on AMH reference values [43].

Besides climatic factors, AMH reference values are also influenced by air quality. In our study, we found a negative correlation between AMH reference values and six air pollution indicators: AQI, PM_2.5_, PM_10_, SO_2_, CO and NO_2_. Previous studies have shown that exposure to ambient air pollutants can have an impact on the reproductive system, and several recent studies have suggested that there may be a strong correlation between female reproductive hormone levels and air pollution [44, 45]. It was observed in an animal experiment that rats exposed to medium (40 mg/mL) and high (80 mg/mL) doses of PM_2.5_ suffered a reduction in anti-Mullerian hormone (AMH) levels [46]. Moreover. Lin et al. found that air pollutants have anti-androgen-like effects in vitro, which may lead to androgen excess through insulin resistance, thus leading to the occurrence of polycystic ovary syndrome and ultimately having an impact on AMH reference values [47]. Several animal toxicology studies have shown that SO_2_ has reproductive toxicity and could indirectly affect AMH reference levels by influencing the level of oxidative stress in the body and affecting hormone secretion in the ovaries [48]. Overall, exposure to ambient air pollutants can affect reproductive hormone levels in body plasma and even have an impact on ovarian function. As mentioned earlier, the secretion of hormone levels plays a key role in the normal development of the reproductive system and changes in hormone levels play a key role in the diagnosis of reproductive endocrine disorders. Most of the previous studies were conducted around animal experiments, lacking direct correlation experiments on AMH reference values in humans, and our study exactly complements this deficiency.

Additionally, AMH reference values were also found to be correlated with soil factors in our study. Soil is the material basis for human survival and the central link in the ecosystem for material exchange and material cycling. It supplies food and vegetables directly to humans by supporting plant growth [49, 50]. The development of modern industry and agriculture has caused a large amount of industrial waste, chemical fertilizers and pesticides to enter the soil, resulting in soil pollution, which affects the quality of agricultural products and human health. It has been proved that soil pollution damages female ovarian function [51]. In regions with more Percentage of silt in topsoil, Percentage of gravel in topsoil, soil particle size is larger, the adsorption of pollutants is poor, pollutants will soon seep under water, and then cause groundwater pollution, and eventually, groundwater enters the life cycle to affect the health of the organism. The total capacity of topsoil is the threshold of soil pollution tolerance, which represents the maximum load of pollutants that the soil can hold. The amount of pollutants that can be contained in the soil environment has an indirect impact on the female body. The alkalinity of topsoil can change the chemical forms of some soil pollutants, and then change their biological toxicity intensity.

In the field of reproductive medicine, the female AMH reference value is a crucial research topic. Our study is interesting and meaningful since it lies at the crossroads of various disciplines by using a variety of geographic disciplinary methodologies. First, since there weren’t many studies on the impact of regional environmental factors like climate, soil, and air quality on AMH, we used correlation analysis to fill this gap. Second, we built a model using the pertinent geographic factors. It is able to generate a reference value for AMH in a region when the geographic environmental factors of that region are understood. Third, kriging interpolation was used to display the high and low values of the AMH reference values on the map using various colors, which will make it easier to further analyze the variations in the spatial distribution of the AMH reference values.

This study still includes Some shortcomings that need to be improved in the future. First, we neglected to take into account the impact of physical activity and some specific pollutants on serum AMH, which would have introduced unrecoverable errors to the results, when choosing demographic characteristics and environmental factors. Second, we solely used national cross-sectional research and testing-related environmental data. The study could not calculate the short-term effect in terms of time since it did not account for the environmental lag of one season or more, which may have led to errors. In order to more effectively control confounding variables, future research will need to include cohort data to analyze the time lag and evaluate eating habits and activity status through questionnaires.

## Conclusions

The reference value of AMH in healthy females of childbearing age is related to 16 geographical factors. The reference value of AMH in various places can be predicted using the ridge regression model developed in this work.

If the latitude, Annual mean temperature, Annual mean relative humidity, Annual precipitation, Annual temperature range, AQI, PM_2.5_, PM_10_, SO_2_, CO, NO_2_, Total capacity of topsoil, Percentage of silt in topsoil, Calcium sulfate content of topsoil, Percentage of gravel in topsoil and the alkalinity of topsoil are known in a certain area. According to the equation:

$$\widehat Y = 7.6849-0.0144X_{1}-0.0006X_{2}-0.02733X_{3}+0.0003X_{4}-0.01292X_{5}-0.0072X_{6}-0.0091X_{7}-0.0042X_{8}-0.00357X_{9}-0.04021X_{10}-0.02080X_{11}-0.75010X_{12}+0.00963X_{13}-0.89583X_{14}-0.00499X_{15}-0.00661\pm 1.82035$$

The AMH reference value can be predicted.

There are regional differences in serum AMH reference values in Chinese females of childbearing age, with lower values in the northwest and northern regions. It is recommended that these differences be taken into account in clinical diagnosis.

### Supplementary Information

**Additional file 1.** Data sources article titles.

## Data Availability

The AMH data that support the findings of this study are openly available in China national knowledge infrastructure (CNKI), Wanfang Scientific Journal Full-text Database, and Pub Med Database. They are available from the published literature for these databases. The titles of this literature are in the Appendix. The location indicators came from the National Bureau of Surveying and Mapping. The following supporting information can be downloaded at http://www.nasg.gov.cn/. The climate indicators were selected from the China Meteorological Science Data Sharing Service Network (http://cdc.cma.gov.cn/). The soil indicators are derived from the Harmonized World Soil Database (HWSD) (http://www.fao.org/nr/land/soils/harmonized-world-soil-database/zh/). The environmental factors data were obtained from the China Meteorological Data Sharing Service System and Environmental Monitoring of China. The data acquisition requires an application. However, the datasets used and/or analyzed during the current study are available from the corresponding author on reasonable request.

## Abbreviations

AMH: Anti-mullerian hormone
BMI: Body Mass Index
CO: Carbon monoxide
O_3_: Ozone
NO_2_: Nitrogen dioxide
SO_2_: Sulfur dioxide
PM_10_: Particulate matter with particle size below 10 μm
PM_2.5_: Particulate matter with particle size below 2.5 μm
GDP: Gross Domestic Product
