# Supplementary material for: Spatial distribution of anti-mullerian hormone in females of childbearing age in China under the influence of geographical environmental factors
Source: BMC Public Health. 2023 Aug 18;23:1571. doi: 10.1186/s12889-023-16431-y (PMC10436535; doi:10.1186/s12889-023-16431-y)
Supplement: Supplementary file 1 — Additional file 1. Data sources article titles. [file 12889_2023_16431_MOESM1_ESM.docx]

1. Yang X, Wang F, Wang F, et al. Clinical significance of serum anti-Mullerian hormone in diagnosis of premature ovarian failure. Laboratory Medicine and Clinic. 2010; 7: 51-52.
2. Yang S, Wang R, Huang X, et al. Effect of anti-Mullerian hormone on ovarian reserve function in trophoblastic tumor patients undergoing chemotherapy. Maternal and Child Health in China. 2010; 25: 2095-2097.
3. Qin X, Yan Y, Shi Q, et al. Clinical value of serum anti-Mullerian hormone in predicting premature ovarian failure. Chinese Journal of Integrated Traditional and Western Medicine. 2010; 19: 3240-3241.
4. Qin X, Yan Y, Shi Q, et al. The relationship between serum anti-mullerian hormone levels and female reproductive dysfunction. Chinese Journal of Integrated Traditional and Western Medicine. 2010; 20: 3644-3646.
5. Qin X. Study on the relationship between serum anti-Mullerian hormone level and polycystic ovary syndrome. Chinese Journal of Experimental Diagnostics. 2011; 15: 1389-1390.
6. Li M, Zhao Y, Ci C, et al. Clinical significance of anti-Mullerian hormone in female systemic lupus erythematosus and its relationship with anti-ovarian cell antibodies. Chinese Journal of Rheumatology. 2010; 305-307.
7. Hou Q, Yan Y. Clinical significance of serum anti-mullerian hormone levels in polycystic ovary syndrome. International Journal of Laboratory Medicine. 2013; 34: 9-10+13.
8. Cui X, Liao W, Mai X. Clinical significance of AMH combined with B-ultrasound in the diagnosis of polycystic ovary syndrome. China Maternal and Child Health Care. 2011; 26: 2203-2205
9. Chen. X, Hong Y, Yang D. Diagnosis and treatment of adolescent polycystic ovary syndrome. International Journal of Reproductive Health/Family Planning. 2013; 32: 330-334.
10. Zou X. Study on influencing factors quality of life correlation of sex hormone levels and distribution of TCM syndrome types in early-onset ovarian insufficiency. Master Chengdu University of TCM, Chengdu. 2020.
11. Zhu P, Ye L, Zhu. Y, et al. Clinical analysis of anti-Mullerian hormone combined with sex hormone test in predicting ovarian reserve function. Contemporary medical. 2017; 23: 91-92.
12. Zhu L. Correlation between primary ovarian insufficiency and serum vitamin D level. Master Zhejiang University Zhejiang. 018.
13. Zhou Y. Clinical effect of kidney-Yuyin decoction combined with acupoint application in the treatment of menopausal syndrome with kidney Yin deficiency. Master Jiangxi University of Traditional Chinese Medicine Jiangxi. 2019.
14. Zhou Y. Effect of hysterectomy on ovarian reserve function. Master Chongqing Medical University Chongqing. 2019.
15. Zhou W, Li X. Relationship between anti-Mullerian hormone and insulin resistance in non-obese polycystic ovary syndrome. International Journal of Obstetrics and Gynecology. 2017; 44: 118-120.
16. Zhou Q, Dong S, Zhang Y, et al.Differences in anti-Mullerian hormone levels among women with different ovarian reserve function. Chinese Journal of Eugenics and Genetics. 2016; 24: 115-117.
17. Zhou N, Yuan J, Wu B, et al. Value of AMH, E_2: LH and FSH levels in evaluating ovarian function status in female infertility patients. Internal medicine. 2020; 15: 42-44.
18. Zhou L, Zheng Y. Analysis of INHB, AMH and endocrine indexes in patients with PCOS. China Maternal and Child Health. 2019; 34: 4419-4421.
19. Zhong X. Ovarian function in. 35-40 years old patients with polycystic ovary syndrome. China Journal of Modern Medicine. 2014; 24: 75-79
20. Zhen Y. Clinical study of Ancient Han health-preserving Essence tablets in the treatment of Qi deficiency and sub-health. Master Hunan University of Traditional Chinese Medicine Hunan. 2019.
21. Zhao Y, Wang L, Wei T, et al. Effect of different hemostatic methods on ovarian reserve function in laparoscopic mature ovarian teratoma stripping. Chinese Journal of Obstetrics and Gynecology. 2017; 52: 57-60.
22. Zhao R. Effect of different hemostatic methods on ovarian reserve function in laparoscopic mature ovarian teratoma stripping. Master Anhui Medical University Anhui. 2016.
23. Zhao M, Sun D, Liu P, et al. Evaluation of ovarian function in. 35-40 year old patients with polycystic ovary syndrome. Journal of Hebei Medical University. 2016; 37: 841-843.
24. Zhang Y, Yang T, Ni W. Diagnostic value of anti-Mullerian hormone IL-21 combined with sex hormone in predicting premature ovarian failure. Journal of Hunan University of Traditional Chinese Medicine. 2017; 37: 1110-1112.
25. Zhang Y, Luo T. Application of anti-Mullerian hormone in children with cryptorchidism. Medicine and Clinic in China. 2019; 19: 3225-3226.
26. Zhang Y. Effect of mifepristone on uterine fibroids and its effect on serum sex hormone. Master Zunyi Medical University Zunyi. 2017.
27. Zhang Y, Li H, Lu J. Effect of coenzyme Q_(10) combined with Jinfeng pill on ovarian function and IVF-ET outcome in elderly pregnant women. Chinese Journal of Medical Frontiers. 2020; 12: 92-96.
28. Zhang Y, Cheng X. Value of serum creatine kinase progesterone and anti-Mullerian hormone in the diagnosis and evaluation of tubal pregnancy. Journal of Clinical Military Medicine. 2019; 47: 683-685.
29. Zhang X, Hao L, Huang R, et al. Correlation between anti-Mullerian hormone and LH/FSH in patients with polycystic ovary syndrome. Journal of Clinical Laboratory Science. 2018; 7: 652-653.
30. Zhang Q, Yuan C, Ying C. Application of serum AMH and inhibin B in the diagnosis of polycystic ovary syndrome. Chinese Journal of Laboratory Medicine. 2019; 652-656.
31. Zhang P, Zhang X. Correlation between serum AMH, visfatin irisin levels and HOMA-IR index in patients with polycystic ovary syndrome and its clinical significance. China Experimental Diagnostics. 2018; 22: 73-74.
32. Zhang L, Yuan Y, Yan L, et al. Diagnostic significance of serum anti-Mullerian hormone in polycystic ovary syndrome. Journal of North Sichuan Medical College. 2019; 34: 635-638
33. Zhang J, Xu Z, Dong R, et al. The value of serum anti-Mullerian hormone and follicle-stimulating hormone levels in the diagnosis of polycystic ovary syndrome. Journal of Guizhou Medical University. 2020; 45: 1337-1340+1346.
34. Zhang J, Liu W, Lian Y. Correlation between endometrial thickness and serum AMH, glucose and lipid metabolism in patients with PCOS. Journal of Molecular Diagnosis and Therapeutics. 2021; 13: 17-20.
35. Zhang G. Correlation between AMH and endocrine and metabolic abnormalities and ART outcomes in PCOS. Master Ningxia Medical University Ningxia. 2017.
36. Zhan Z, Cao M, Wang H, et al. Effect of AMH, FSH, LH combined with gynecological color Doppler ultrasound in the early diagnosis of premature ovarian failure. Contemporary medical 2019; 25: 6-8.
37. Zhan F, Liang Y. Correlation between fetal growth restriction and adult ovarian reserve function. Chinese and Foreign Women's Health Research. 2018; 88-89.
38. Yuan L, Cheng M, Liu X, et al. Status of serum AMH levels in healthy women of childbearing age in Shenzhen and its value in the diagnosis and treatment of PCOS and POF. Journal of Modern Laboratory Medicine. 2017; 32: 141-144.
39. Yu Y, Liu X, Yao S. Relationship between serum anti-Mullerian hormone and luteinizing hormone levels and the efficacy of ovulation induction in infertile patients with polycystic ovary syndrome. Journal of Lanzhou University (Medical Science) 2019; 45: 10-13+19.
40. Yu Y, Ge Q, Yu X, et al. Correlation between serum anti-mullerian hormone and clinical endocrine indicators in polycystic ovary syndrome. Journal of Bengbu Medical College. 2018; 43: 730-733.
41. Yang Y, Li Y, Wang L, et al. The significance of serum anti-Mullerian hormone in the diagnosis of polycystic ovary syndrome. A Collection of Contemporary medical articles. 2019; 17: 161-162.
42. Yang H. Effect of acupuncture on ovarian function in patients with early-onset ovarian insufficiency: a case registration study. Master China Academy of Chinese Medical Sciences Beijin. 2019.
43. Yang H, Shi Y, Wang Q, et al. Correlation analysis of INH-B, Leptin AMH and sex hormones in patients with polycystic ovary syndrome. Chinese Journal of Family Planning 2020; 28: 1018-1021.
44. Xu Y, Huang Y. Evaluation of ovarian reserve and diagnostic value of AMH in patients with PCOS. International Journal of Reproductive Health/Family Planning. 2019; 38: 230-235.
45. Xu L, Xu H, Meng Y, et al. Correlation analysis of serum anti-mullerian hormone and free testosterone levels in patients with polycystic ovary syndrome. World Clinical Drug. 2019; 40: 255-260.
46. Jun X, Wu J. Transvaginal three-dimensional ultrasound combined with anti-Mullerian hormone in the evaluation of ovarian function in polycystic ovary syndrome. China Maternal and Child Health Care. 2019; 34: 3100-3103.
47. Xu J, Wang Y, Jiang G, et al. Clinical application of serum anti-Mullerian hormone detection in polycystic ovary syndrome. International Journal of Lab Medicine. 2017; 38: 35-36.
48. Xiong M, Zhu H, Luo Y, et al. Researh material - 11. Zhu J P, Effect of ethinylestradiol cyproterone tablets on serum anti-Mullerian hormone in patients with PCOS. Progress in Modern Obstetrics and Gynecology. 2018; 27: 195-197.
49. Xing Y. Effects of exogenous and endogenous TSH inhibition on serum anti-Mullerian hormone. Master China Medical UniversityBeijin. 2019.
50. Xing J, Zhang F. Correlation between serum anti-Mullerian hormone and free testosterone in patients with polycystic ovary syndrome. Journal of Henan Medical College. 2020; 32: 117-119.
51. Xie Q, HongY, Chen C, et al. Effect of laparoscopic cyst removal on serum anti-mullerian hormone levels in patients with benign ovarian cysts. Chinese Maternal and Child Health. 2013; 28: 4630-4634.
52. Xie P, Long L, Han L. Application of anti-Mullerian hormone combined with ovarian interstitial artery blood flow index and ultrasound index in the analysis of ovarian reserve function. Chinese Medical Innovation. 2019; 16: 138-141.
53. Xiao J, Wang B, Tao S. Combined application of AMH, FSH/LH and gynecological ultrasound in the early diagnosis of premature ovarian failure. Current Medicine. 2019; 25: 147-148.
54. Xiao H, Zhai Q, Lu R, et al. Serum AMH levels in healthy women of childbearing age in Bao 'an district and its application in the diagnosis and treatment of PCOS and POF. Clinical Blood Transfusion and Laboratory Tests. 2018; 20: 279-282.57.
55. Xiang G, An M, Zhao H, et al. Clinical value of anti-Mullerian hormone in the diagnosis of polycystic ovary syndrome. Chinese Journal of Health Laboratory Science. 2018; 28: 71-73.
56. Xi Y, Shen Z, Bai H. The expression levels of IL-17A, Betatrophin and soluble CD68 in patients with PCOS and their relationship with ovarian function. Journal of Molecular Diagnosis and Therapy. 2020; 12: 1336-1340.
57. Wu M, Lou H, Liu F. Changes of serum IL-21 and AMH levels in patients with premature ovarian failure and their diagnostic value. Chinese Journal of Family Planning. 2020; 28: 847-849.
58. Wu J. Correlation analysis of anti-Mullerian hormone and polycystic ovary syndrome. Chinese Journal of PharmacoEconomics. 2016; 11: 100-102.
59. Wu Y, He Z, Lin B. Study on predicting premature ovarian failure and perimenopause by AMH. Clinical Medical Engineering. 2018; 25: 745-746.
60. Wu X, Chen X, Mo Y, et al. Clinical value of anti-Mullerian hormone in evaluating ovarian reserve decline. Heilongjiang Medicine. 2021; 45: 57-59.
61. Wu T, Weng M. Changes of serum anti-mullerian hormone levels in girls with idiopathic central precocious puberty. Chinese Journal of Disability Medicine. 2014; 22: 195-196.
62. Wu L, Yan L. Evaluation of ovarian reserve capacity before and after laparoscopic ovarian cyst stripping by three indicators. Laboratory Medicine & Clinic. 2018; 15: 890-892+896.
63. Wu J. The significance of AMH in the selection of pregnancy assistance program for patients with ovarian dysfunction. Master Wannan Medical College Wannan. 2020
64. Weng G, Sun Z, Li Q. Value of serum anti-Mullerian hormone (AMH) in diagnosis of polycystic ovary syndrome. China Medical Sciences. 2018; 8: 114-116.
65. Wei D, Li P, Huang X. Application value of anti-Mullerian hormone combined with sex hormone in evaluation of ovarian reserve function. Clinical medical research and practice. 2020; 5: 119-120.
66. Wang Z, Tong J. Analysis of the changes of FSH, LH, E2 and AMH levels in patients after myomectomy. Modern Medicine. 2018; 46: 197-200.
67. Wang S, Zeng J, Lang J, et al. The characteristics of traditional Chinese medicine constitution distribution of perimenopausal osteopenia and its correlation with serum anti-Mullerian hormone and estradiol. Chinese Folk Therapy. 2020; 28: 69-70.
68. Wang Q, Sun J: Li X. Detection of serum anti-Mullerian hormone and study on eugenics and eugenics. Marker immunoassay and Clinic. 2016; 23: 1099-1100.
69. Wang N. Clinical effect of laparoscopic myomectomy in the treatment of uterine fibroids. Clinical Medicine Research and Practice. 2020; 5: 87-89.
70. Wang L. Characteristics and clinical significance of serum AMH monitoring ovarian reserve function in healthy and reproductive age women with systemic lupus erythematosus. Zhejiang Trauma Surgery. 2020; 25: 217-219.
71. Wang L, Ruan X, Cui Y, et al. Analysis of the characteristics of serum anti-mullerian hormone levels in different phenotypes of polycystic ovary syndrome. Journal of Capital University of Medical Science. 2016; 37: 444-448.
72. Wang K, Zhu G, Chen J, et al. Expression of AMH in serum and ovarian tissue of patients with ovarian endometriotic cyst. Hainan Med. 2020; 31: 689-692.
73. Wang J. Correlation analysis of sex hormones blood lipids body mass index and anti-Mullerian hormone abnormalities in patients with polycystic ovary syndrome. Chinese Contemporary Medicine. 2020; 27: 190-193.
74. Wang H, RuanX, Li Y, et al. Exploration of the relationship between malondialdehyde retinol and the pathogenesis of polycystic ovary syndrome. Journal of Capital Medical University. 2018; 39: 512-516.
75. Tang L. Clinical predictive value of anti-Mullerian hormone and inhibin B in polycystic ovary syndrome. Chinese Community Physicians. 2019; 35: 69+73.
76. Tang J, Wu S, Xu Z, et al. The diagnostic value of serum anti-Mullerian hormone level in patients with polycystic ovary syndrome. Journal of Tropical Medicine. 2019; 19: 1386-1388.
77. Tang H. Differential analysis of AMH expression in serum of patients with polycystic ovary syndrome and low ovarian reserve. Journal of Clinical Laboratory Science. 2018; 7: 34-35.
78. Tan J. AMH, LH, FSH in the diagnosis of polycystic ovary syndrome. Electronic Journal of Practical Gynecological Endocrinology. 2020; 7: 122+130.
79. Sun Z. Subtype changes of peripheral blood mononuclear cells in patients with polycystic ovary syndrome. Master China Medical University Beijin. 2019.
80. Shi Q. Study on diagnostic markers of male infertility and its BPNN diagnostic model. Master Huazhong University of Science and Technology Wuhan. 2020.
81. Shi X, Shi X, Zhou Q, et al. Application analysis of anti-Mullerian hormone in evaluation of female ovarian function. China Maternal and Child Health Care. 2019; 34: 4512-4514.
82. Shen C, Shi X, Qiu Y, et al. Correlation between serum FAI and AMH and IR in patients with polycystic ovary syndrome. Preventive Medicine. 2018; 30: 467-470.
83. Shen S, Liu K. Predictive value of serum anti-Mullerian hormone (AMH) in patients with premature ovarian failure. Trace Elements and Health Research. 2017; 34: 28-29.
84. Peng C, Ma H, Liu S. Clinical application of anti-Mullerian hormone measurement in predicting ovarian reserve function and premature ovarian failure in women of reproductive age in plateau area. Plateau Medicine. 2018; 28: 47-49.
85. Pang Y. Mullerian hormone test in the diagnosis of polycystic ovary syndrome. Chinese and Foreign Medical Research. 2018; 16: 54-56.
86. Pan Jing: Wang Yanmei: Bo Xiaoli, Uygur and Han ethnic groups of anti-mullerian duct hormone levels and their correlation with age. Prescription drugs in China 2019; 17:138-140.
87. Pan J, Bo X. Anti-Mullerian hormone levels in Uygur and Han populations with polycystic ovary syndrome and its correlation with age. International Journal of Laboratory Medicine. 2019; 17: 138-140.
88. Nie C, Lu F, Huang Q. Clinical value of serum anti-Mullerian hormone and sex hormone detection in the diagnosis and prediction of premature ovarian failure. Shenzhen Journal of Integrated Traditional Chinese and Western Medicine. 2016; 26: 87-88.
89. Mu C. Effect of different methods of total hysterectomy combined with salpingectomy on ovarian function. Master Hebei North University Hebei. 2016.
90. Mo N. Risk of ovarian reserve decline in patients with renal deficiency rheumatoid arthritis. Master Guangzhou University of Traditional Chinese Medicine Guangzhou. 2016.
91. Meng W, Peng L, Liu X. Correlation between premature ovarian failure and the expression of AMH and FMR1 genes. Chinese Journal of Eugenics and Genetics. 2018; 26: 18-20.
92. Ma F, Wang H. Analysis of anti-Mullerian hormone anti-endometrial antibody and anti-ovarian antibody in female infertile patients. Chinese Journal of Eugenics and Genetics. 2016; 24: 106-107.
93. Luo F, Gu D, Hua W. AMH, INHB and sex hormone combined detection to evaluate ovarian reserve function before and after gynecological laparoscopic surgery. Chinese Modern Drug Application. 2020; 14: 29-30.
94. Lu K. Clinical efficacy and analysis of three different surgical methods in the treatment of adenomyosis. Master Guangxi University of Traditional Chinese Medicine Nannin. 2017.
95. Lu F, Nie C. Application of AMH FSH combined with ultrasonography in predicting premature ovarian failure. Forum on Primary Medicine. 2020; 24: 957-959.
96. Liu Z. Clinical study of anti-Mullerian hormone AMH in diagnosis of polycystic ovary syndrome. Chinese and Foreign Women's Health Research. 2019; 61-62.
97. Liu Y, Zhang X. Analysis of serum anti-mullerian hormone in patients with polycystic ovary syndrome and chocolate cyst of ovary. Aerospace Medicine Journal. 2018; 29: 1314-1315.
98. Liu Y. The diagnostic value of serum anti-Mullerian hormone level in premature ovarian failure. Henan Medical Research. 2019; 28: 991-993.
99. Liu X, Zeng J, Liu W, et al. Research progress of anti-Mullerian hormone in the diagnosis of polycystic ovary syndrome. Chinese Journal of Reproduction and Contraception. 2020; 40: 1026-1030.
100. Liu X, Guan X, Wang Y. Application of serum hormone measurement combined with vaginal color ultrasound in early diagnosis of premature ovarian failure. Chinese and Foreign Women's Health Research. 2020; 71-72.
101. Liu Qian: Xie Jianglin: Wang Xizi: Mona: Li Chenchen: Zhang Mingying: Liu Lijuan: Chen Guangxing, a study on the decline of ovarian reserve function of renal deficiency systemic lupus erythematosus, the 16th Chinese Rheumatology Annual Conference of Integrated Traditional Chinese and Western Medicine, Guangzhou, China, 2018; Guangzhou, Guangdong, China, 2018; pp. 202-203.
102. Liu X, Xie J, Wang X, et al. Decreased ovarian reserve in patients with renal deficiency systemic lupus erythematosus. Journal of Guangzhou University of Traditional Chinese Medicine. 2018; 35: 575-580.
103. Liu P. Xun Q. Clinical value of anti-Mullerian hormone combined with sex hormone in the diagnosis of polycystic ovary syndrome. Chinese Primary Medicine. 2020; 27: 1979-1983.
104. Liu J, Yuan Y, Zhang W, et al. Relationship between serum resist in AMH, SHBG and insulin resistance in patients with PCOS. Laboratory & Laboratory Medicine. 2020; 38: 502-503+515.
105. Liu N. Analysis of serum AMH, FSH, LH and E_2 levels in. 170 female infertile patients. International Medical and Health Review. 2017; 23: 3079-3081.
106. Lin T, Chen H, Ye G, et al. Application of serum anti-Mullerian hormone combined with basal endocrine hormone detection in predicting early-onset ovarian insufficiency. Journal of Practical Medical Technology. 2019; 26: 711-712.
107. Lin Rong. ~ (131) A prospective study of the effect of I treatment on ovarian storage function in patients with differentiated thyroid cancer. Master degree, Fujian Medical University, 2019.
108. Liang R. Serum anti-Mullerian hormone interleukin-21 combined with sex hormone detection in the diagnosis of premature ovarian failure. Modern Diagnosis & Therapy. 2020; 31: 3288-3289.
109. Liang G, Zheng W, Liu C, et al. The relationship between endocrine and metabolic abnormalities and AMH levels in patients with PCOS. Chin J Med Sci. 2018; 8: 254-256.
110. Liang, D. Relationship between AMH and INHB and sex hormones in patients with polycystic ovary syndrome and their application value. Laboratory Medicine and Clinic. 2017; 14: 2245-2247.
111. Li Z, Lai G, Zhao Y. Analysis of anti-Mullerian hormone in polycystic ovary syndrome and endometriosis. Chinese Journal of Practical Gynecology & Obstetrics. 2017; 33: 641-643.
112. Li Y, Shen J. Analysis of influencing factors of atrophic vaginitis. Jiangsu Medicine. 2020; 46: 1034-1037.
113. Li Y, Wang J. Detection and significance of sex hormones thyroid function and immune antibodies in infertility patients with PCOS. International Journal of Laboratory Medicine. 2020; 41: 1210-1213.
114. Li X, Wu H, Tan J. Application of anti-Mullerian hormone in diagnosis and clinical management of polycystic ovary syndrome. Journal of Practical Obstetrics and Gynecology. 2015; 31: 567-572.
115. Li P, Tan Z, Xie X, et al. AMH and INHB for the diagnosis of polycystic ovary syndrome and their efficacy evaluation. Chinese Journal of Laboratory Medicine. 2017; 40: 391-395.
116. Li L, Li X. Correlation between serum AMH level and insulin resistance and androgen in patients with PCOS. Chinese and Foreign Medical Research. 2020; 18: 140-142.
117. Li J, Zhang F, Effect of Tiaojing Yiyu prescription on low ovarian reserve and thin endometrium. Journal of Guangzhou University of Traditional Chinese Medicine. 2021; 38: 36-41.
118. Li H, Zhou Y, Jiang Y, et al. Application value of anti-Mullerian hormone in patients with polycystic ovary syndrome. China Maternal and Child Health. 2020; 35: 2476-2479.
119. Li H, Guo Z, Yin Z, et al. Evaluation of anti-Millerian hormone for individualized treatment of ovarian function inhibition in young breast cancer patients. China Oncology. 2015; 25: 983-988.
120. Li D, Zhang L. Correlation between endometrial thickness and serum AMH and glucose and lipid metabolism indexes in patients with polycystic ovary syndrome. Clinical Medicine Research and Practice. 2020; 5: 131-133.
121. Jing X, Xu L, Chen B, et al. Polymorphism analysis of AMH, AMHR-Ⅱ gene in idiopathic premature ovarian failure. China Medicine and Pharmacy. 2017; 7: 7-10.
122. Jin J, Ruan X, Hua L, et al. Occurrence of polycystic ovary syndrome with low ovarian reserve function. Journal of Reproductive Medicine. 2018; 27: 103-107.
123. Jiang X, Ding C, Chen X, et al. Anti-Mullerian hormone and its correlation with insulin resistance and ovarian reserve ability in patients with polycystic ovary syndrome. Chinese Journal of Health Laboratory Science. 2021; 31: 53-55.
124. Jiang N, Cheng X, Wang X. Clinical value of AMH, LH, T in the diagnosis of PCOS and its relationship with insulin resistance. Chinese Journal of Family Planning. 2020; 28: 1609-1612+1723.
125. Ji X, Liang X, Ma J. Correlation between ribosomal protein gene S26 and endocrine hormone levels in patients with polycystic ovary syndrome. Family Planning and Obstetrics and Gynecology of China. 2018; 10: 49-52.
126. Huang J, Liu M, Zhang T, et al. Correlation between anti-Mullerian hormone and insulin resistance and sex hormones in non-obese polycystic ovary syndrome. Journal of Reproductive Medicine. 2020; 29: 949-952.
127. Huang C, Han K. Relationship between serum AMH, estradiol and antral follicle number levels and endometrial receptivity after ovarian endometriotic cyst stripping. Southeast National Defense Medicine. 2020; 22: 501-504.
128. Hu L, Wang H, Xie Q, et al. Effect of different surgical methods on ovarian function in uterine fibroids. Chinese Journal of Practical Medicine. 2017; 12: 23-25.
129. Hu B. Anti-Mullerian hormone and endocrine metabolic indicators in the diagnosis of polycystic ovary syndrome. Current Medicine. 2020; 26: 153-154.
130. Hou S, Cheng W, Shen Q, et al. Changes of AMH and sex hormone levels and their diagnostic value in patients with different ovarian reserve function. International Journal of Laboratory Medicine. 2020; 41: 1483-1486.
131. He X, Zhuang J, Lv L, et al. Clinical significance of anti-Mullerian hormone test in patients with systemic lupus erythematosus. Heilongjiang Med Heilongjiang Medicine And Pharmacy. 2018; 42: 245-246+248.
132. Ge L. The predictive value of AMH and LH/FSH in patients with polycystic ovary syndrome. Chinese and Foreign Medical Research. 2021; 19: 88-90.
133. Ge J. The application of serum AMH combined with sex hormone and inhibin B in the diagnosis of polycystic ovary syndrome. Journal of Baotou Medical College. 2020; 36: 14-17+37.
134. Gao H, Ma J, Wang X, et al. Ovarian reserve function in patients with systemic lupus erythematosus. China Medical Review. 2016; 13: 125-128.
135. Feng L, Deng M, Li X, et al. Correlation between anti-mullerian hormone levels and insulin resistance and reproductive hormone levels in patients with PCOS. Journal of Reproductive Medicine. 2021; 30: 30-34.
136. Feng J. Study on the characteristics of ovarian three-dimensional ultrasound parameters and their correlation with hormone levels in polycystic ovary syndrome. Master Guangxi Medical University Nannin. 2020.
137. Fan Y, Xie J, Shi R, et al. Anti-Mullerian hormone and follicle-stimulating hormone in the diagnosis of polycystic ovary syndrome. Chinese Journal of Clinical Pharmacology. 2019; 35: 2665-2667.
138. Du J, Li J, YanY, et al. Detection and clinical application of serum AMH,LH and FSH levels in patients with polycystic ovary syndrome. Current Journal of Laboratory Medicine. 2019; 34: 68-71.
139. Dong Z, Xie X, Mao X, et al. Research progress on basal levels of anti-Mullerian hormone in endometriosis patients. International Journal of Obstetrics and Gynecology. 2017; 44: 228-231+243.
140. Dong Y, Lou Y. The diagnostic significance of anti-Mullerian hormone in polycystic ovary syndrome in plateau area. Qinghai Journal of Medicine. 2020; 50: 10-12.
141. Ding J, Shao J, Jian H, et al. Anti-Mullerian hormone (AMH) and polycystic ovary syndrome: a clinical study. Current Medicine. 2018; 24: 93-95.
142. Zhai Q, Liu Q, Lu J, et al. Serum levels of AMH, TNF-α, T and DHEA-S in patients with polycystic ovary syndrome and their clinical significance. Hainan Medical College. 2020; 31: 1100-1102.
143. Deng Y, Liu C, Yin Z, et al. Serum levels of IL-33: sST2 and AMH in patients with polycystic ovary syndrome and their clinical significance. China Medicine and Pharmacy. 2019; 9: 122-125.
144. Deng W, Jiang L, Zhao N, et al. Anti-Mullerian hormone in the diagnosis of polycystic ovary syndrome. Clinical Medicine Practice. 2020; 29: 439-441.
145. Dai L, Sun Y. Anti-Mullerian hormone inhibin B and homeostasis model insulin resistance index in patients with polycystic ovary syndrome and their diagnostic value. Chinese Journal of Health Laboratory Science. 2020; 30: 1482-1484.
146. Chen Y, Yue C, Ying C. Correlation analysis of anti-Mullerian hormone with different phenotypes of polycystic ovary syndrome. Chinese Journal of Reproduction and Contraception. 2017; 37: 433-436.
147. Chen Y, Zhang Q, Yue Z. Application of anti-Mullerian hormone and endocrine metabolic index in the diagnosis of polycystic ovary syndrome. Chinese Journal of Reproduction and Contraception. 2018; 38: 847-851.
148. Xiu Q, Ma X, Zhong J, et al. Anti-Mullerian hormone and embryo prognosis in women with natural conception. Chinese Journal of Health Medicine. 2019; 21: 275-276.
149. Chen T, Peng S, Luo D. The efficacy of laparoscopic myomectomy in the treatment of uterine fibroids and its effect on antimueller hormone and local cervical microcirculation. Hebei Medicine. 2019; 25: 322-325.
150. Chen H. Effect of different surgical methods on ovarian reserve function in tubal pregnancy patients. Master Bengbu Medical College Benbu. 2017.
151. Chen H, Li B, Wang T. Predictive value of serum anti-Mullerian hormone in patients with premature ovarian failure. Hainan Mediai. 2018; 29: 3274-3276.
152. Zeng J, Yin J, Wang G, et al. Column Lin kun: Tang D E, Application value of serum AMH detection in polycystic ovary syndrome. International Journal of Laboratory Medicine. 2019; 40: 1070-1072.
153. Cao S, Hu Y. Effects of advanced glycosylation end products serum anti-Mullerian hormone and inhibin B on reproductive organ development in children with type. 1 diabetes mellitus. Journal of Medical Research. 2017; 46: 116-118+128.
154. Cao S, Shan X, Hu Y. Effect of unilateral cryptorchidism on serum anti-mullerian hormone and inhibin B levels in children. National Journal of Andrology. 2016; 22: 805-808.
155. Cao K, Luo X, Shen D, et al. A preliminary study on the relationship between anti-Mullerian hormone and its type Ⅱ receptor gene polymorphism and cryptorchidism. International Journal of Laboratory Medicine. 2019; 40: 1945-1949.
156. Cao D, Bi X, Shen K. Application prospect of anti-Mullerian hormone in gynecological neoplasms. Journal of Shandong University (Medical Edition) 2018; 56: 8-12.
157. Zhou J, Zhong J, Huang W, et al. Predictive value of AMH, FSH, LH, E_2 combined detection in ovarian reserve function in infertile patients. Smart Health. 2021; 7: 13-15+18.
158. Zhou H, Li Y, Zhang Z, et al. Pathophysiological role and diagnostic value of AMH in PCOS. Chinese Journal of Eugenics and Genetics. 2022; 30: 157-160.
159. Zhong L, Xiao Z, Liu Y, et al. The value of serum anti-Mullerian hormone combined with sex hormone detection in the clinical diagnosis of polycystic ovary syndrome. Chinese Contemporary Medicine. 2022; 29: 115-117+120.
160. Zhao P, Bao A. The diagnostic value of combined detection of serum anti-Mullerian hormone and sex hormone in polycystic ovary syndrome. Journal of Nantong University (Medical Science Edition) 2022; 42: 186-188.
161. Zhao D. Effect of treatment regimen on the outcome of assisted reproduction in ovarian endometriotic cyst. Master Jilin University Changchun. 2022.
162. Zhang Q, Yue C, Wang Z, et al. Distribution of serum AMH levels in different clinical manifestations in women of childbearing age. Chinese Journal of Preventive Medicine. 2022; 56: 985-989.
163. Zhang P, Cao H, Cao J, et al. Relationship between AMH level and estrogen level and pregnancy outcome in patients with polycystic ovarian syndrome. Guizhou Pharmaceutical. 2022; 46: 343-345.
164. Chang L, Wang Y, Hu X. Diagnostic value of transvaginal ultrasonography combined with serum anti-Millerian hormone levels in polycystic ovary syndrome. Medical Equipment. 2021; 34: 43-45.
165. Zhang L, Shi X, Zhu T, et al. Study on hormones related to ovarian function in women aged. 21-30 years in Xi 'an area. Clinical Medicine Research and Practice. 2021; 6: 63-66.
166. Zhang J, Chen L, Huang Z, Liang J. Diagnostic value of AMH, DHEAS and SHBG in polycystic ovary syndrome. China Medical Engineering. 2021; 29: 67-70.
167. Zhang C. Correlation between anti-Mullerian hormone levels and FSH, LH, E_2 levels in premature ovarian failure patients. Chinese Medical Science. 2022; 34: 5-7.
168. Zhang B, Wei W, Yang C. Expression of AMH, DHEA-S, LH and FSH in serum of patients with polycystic ovary syndrome and its clinical significance. Guizhou Pharmaceutical. 2022; 46: 126-127.
169. Yue R. Study on the correlation between AMH level and physical and chemical indexes such as endometrial status and traditional Chinese medicine syndrome type in infertility patients. Master Tianjin University of Traditional Chinese Medicine Tianjin. 2021.
170. Yuan L. Serum AMH, LH, FSH levels and their significance in infertile patients with endocrine dysregulation. Chinese and Foreign Medical Research. 2021; 19: 39-41.
171. Yu R. Application value of AMH detection in the diagnosis of polycystic ovary syndrome. Journal of Rare and Rare Diseases. 2022; 29: 62-63.
172. Ye Z, Chen Ping. Relationship between IL28B-SNP, AMH, Sp17Ab and hepatitis B virus susceptibility in patients with low ovarian response. Chinese Journal of Family Planning. 2022; 30: 375-378.
173. Yang Y, Zhou Z, Yang L, et al. Correlation between rapidly progressing central precocious puberty and serum anti-millerian hormone and inhibin B levels in girls. Journal of Chongqing Medical University. 2022; 47: 263-267.
174. Yang H. Effect factors of acupuncture treatment on premature ovarian insufficiency and machine learning pregnancy prediction. Doctor China Academy of Chinese Medical Sciences Beijin. 2022.
175. Yang F, Zeng F, Qi Y. Correlation between ovarian reserve function and blood glucose metabolism in perimenopausal women. Chinese Journal of Diabetes. 2021; 29: 688-691.
176. Yan L, Pan C, Pei F. Clinical application of laparoscopic core-pulling salpingectomy combined with interstitial sutures and ligation and its effect on ovarian function. China Maternal and Child Health Care. 2021; 36: 1677-1679.
177. Xu Y, Dong H. Anti-Mullerian hormone combined with sex hormone to evaluate ovarian function in American workers. Chinese Modern Doctor. 2021; 59: 64-67.
178. Xu S, Li J, Ma J. Correlation between vitamin D level and ovarian reserve function in infertile women. Journal of Bengbu Medical College. 2022; 47: 1192-1195.
179. Xiong Y, Liu L, Hu X, et al. Application value of AMH, IFN-γ, IL-2 detection in diagnosis and evaluation of premature ovarian failure. PLA Medical Journal. 2022; 34: 63-66.
180. Xiong W, Ge Kuang Z, Zhao F. Economists: Big bug: KuangZhaoZhong: Zhao F G, Analysis of serum levels of luteinizing hormone testosterone and anti-Mullerian hormone in patients with polycystic ovary syndrome. China Maternal and Child Health. 2022; 37: 2022-2024.
181. Xiong L, Zou J, Wang R, et al. Batty. Effect of clomiphene citrate combined with HCG on AMH level and ovulation rate in patients with PCOS. Central South Journal of Medical Science. 2022; 50: 572-575.
182. Xiang D, Wang K, Zhang T, et al. Study on the relationship between the changes of vascular endothelial growth factor CD4~+/CD8~+ levels and sertoli cell function in children with high cryptorchidism before and after surgery. Chinese Journal of Sex Science. 2022; 31: 5-8.
183. Wu S, Yang H, Gong Y, et al. Expression of serum AMH, FSH, LH and E2 levels in infertile patients and their correlation with ovarian function. Journal of Guangdong Medical University. 2021; 39: 338-340.
184. Wu S, Wang L. Correlation of serum anti-Millerian hormone and integrin β1 levels with uterine spiral artery blood flow parameters and vascular endothelial cell injury indicators in patients with preeclampsia in the second and third trimester. Chinese Journal of Family Planning. 2022; 30: 2120-2123.
185. Wu J, Zhao S, Wang L, et al. Value of floating vaccine and serum AMH level in the evaluation of ovarian reserve function in female APS patients of reproductive age. Laboratory Medicine. 2021; 36: 696-699.
186. Wu J, Nie S, Zhao R, et al. Serum INHB, AMH, FSH and LH levels in predicting ovarian reserve decline and evaluation by endovascular ultrasound after ovulation induction therapy. Chinese Journal of Family Planning. 2022; 30: 836-840.
187. Wu H, Zeng B, Huang Q, et al. Serum SF, AMH, APN and NF-κB are associated with insulin resistance and IVF-ET pregnancy outcomes in obese polycystic ovary syndrome. Advances in Modern Biomedicine. 2022; 22: 3153-3157+3142.
188. Wei Z, He S, Li X, et al. Correlation between serum HE4: Anti-CCP and AMH levels and systemic lupus erythematosus. International Journal of Laboratory Medicine. 2021; 42: 1387-1390.
189. Wei X, Zhang C, Li K, et al. Association of anti-Mullerian hormone blood lipid levels and ovarian ultrasonography indicators with endometrial thickness in patients with polycystic ovary syndrome. Modern Medicine and Health. 2022; 38: 364-367.
190. Wei X, Yang H. Effects of progetrienone combined with tripraline acetate on AMH, EMAb and CA125 levels in patients with endometriosis after surgery. Clinical Medicine Research and Practice. 2022; 7: 61-65.
191. Wang Y, Tang L, Wang L, et al. A prospective study of cognitive function changes after total hysterectomy in women of reproductive age. Chinese Journal of Practical Gynecology & Obstetrics. 2021; 37: 757-761.
192. Wang X, Luo L, Xi H, et al. Relationship between serum vitamin B12 level and IVF-ET outcome in patients with high AMH level. Journal of Reproductive Medicine. 2022; 31: 321-326.
193. Wang X, Luo C, Zhu W, et al. Any He kelamayi region the childbearing age women against muller tube reference range for the establishment and the correlation between sex hormone and hormone. International Journal of Laboratory Medicine. 2022; 43: 2156-2160.
194. Wang X, Hu B. Correlation of serum. 25-(OH)D3 and AMH with ovarian responsiveness and their value in predicting pregnancy outcome after frozen-thawed embryo transfer. Chinese Journal of Family Planning. 2022; 30: 614-617+621.
195. Wang Q, Chen P, Deng Y. Effect of ovarian endometriotic cyst stripping on anti-Mullerian hormone serum ferritin and its clinical value analysis. China Maternal and Child Health. 2022; 37: 3528-3531.
196. Wang J, Chen M, Gong S, et al. Establishment of reference intervals for serum anti-Mullerian hormone in children aged. 0-12 years in Hubei province. Journal of Microcirculation. 2021; 31: 63-66.
197. Wang F, Cheng H, Wang D, et al. Correlation between anti-Mullerian hormone level and ovarian responsiveness in women of reproductive age. Journal of Clinical and Experimental Medicine. 2021; 20: 2637-2640.
198. Wan W. Expression and clinical significance of serum anti-Mullerian hormone prolactin and sex hormone in PCOS patients with infertility. Chinese Medical Innovation. 2021; 18: 157-160.
199. Tian H, Wu J, Xu H. Changes and clinical significance of serum IGF-1: DHEAS and AMH levels in fast-progressing adolescent girls. Chinese Journal of Medical Sciences. 2021; 23: 1866-1868.
200. Tian H, Wu D, Ru W, et al. Research progress in clinical treatment of Mullerian duct perpetuation syndrome. Chinese Journal of Urology. 2022; 43: 628-631
201. Teng X, Wang Z, Xu B, et al. Changes of anti-Mullerian hormone and sex hormone levels in perimenopausal women with osteoporosis and their predictive value. China Maternal and Child Health. 2022; 37: 2880-2883.
202. Tang X, Zhou D, Ma Y, et al. Correlation between anti-Mullerian hormone and insulin metabolism in patients with PCOS. Chongqing Medicine. 2022; 51: 1679-1682.
203. Tang X, Peng J, Chen J, et al. Effects of different pregnancy status on serum anti-Mullerian hormone levels in women. Heilongjiang Medicine And Pharmacy. 2022; 46: 585-587.
204. Sun N, Gao S, An L, et al. Correlation between serum inhibin B, anti-Mullerian hormone and sex hormone levels and uterine artery blood flow parameters in patients with premature ovarian failure. Advances in Modern Biomedicine. 2021; 21: 992-995.
205. Sun J. Correlation between serum AMH, SHBG, sex hormones and lipid metabolism in patients with polycystic ovary syndrome. Chinese Medical Science. 2022; 34: 115-117.
206. Sun J, Zeng X, Gao H. Correlation analysis of anti-Mullerian statin B and sex hormone levels with perioperative changes in ovarian reserve function in patients undergoing laparoscopic surgery. China Maternal and Child Health. 2022; 37: 2833-2836.
207. Sun H, Ji X, Su H, et al. The predictive value of anti-Mullerian hormone level on ovarian responsiveness in patients undergoing in vitro fertilization-embryo transfer assisted conception. Chinese Journal of Sex Science. 2021; 30: 54-57.
208. Shou Y, Jin X, Shou H, et al. Anti-Mullerian hormone screening results of reproductive age women with Hashimoto's thyroiditis in Keqiao District of Shaoxing city. Maternal and Child Health in China. 2021; 36: 1272-1274.
209. Ran Y. Discussion on the value of AMH in diagnosis and curative effect evaluation of polycystic ovary syndrome. Master Chongqing Medical University ChongQin. 2022.
210. Qu Z. Diagnostic value of combined detection of serum anti-Mullerian hormone and sex hormone in PCOS. Famous Doctor. 2021; 46-47.
211. Pan M. Value of transvaginal three-dimensional ultrasound combined with AMH in evaluating ovarian reserve function in patients with systemic lupus erythematosus. Master Hangzhou Normal University Hangzhou. 2021.
212. Pan L, Huang Y, Lv J, et al. The relationship between anti-Mullerian hormone level and antral follicle count and oocyte number in patients with in vitro fertilization (IVF). Chinese Journal of Medical Sciences. 2022; 24: 1363-1367.
213. Pyrdan Nasr Nurbia Abra. Effect of serum anti-Mullerian hormone and sex hormone levels on ovulation outcome after ovulation induction therapy in infertile patients with polycystic ovary syndrome. Chinese Journal of Sex Science. 2022; 31: 33-37.
214. Niu J, Qi J, Niu R. Association between vitamin D and ovarian reserve markers in infertile women. Clinical Research. 2022; 30: 1-5.
215. Mbageko Adili. Clinical characteristics of PCOS patients with metabolic diseases in first-degree relatives. Master Xinjiang Medical University Xinjiang. 2021.
216. Mo H, Wang H, Long Y, et al. Reference range analysis of anti-Mullerian hormone in different ethnic women in Yunnan. China Maternal and Child Health. 2022; 37: 1444-1446.
217. Mao X. Measurement and laboratory value of hormone levels in women with different ovarian reserve function. Chinese Community Physicians. 2021; 37: 105-106.
218. Mao S, Qiang Y, Li H, et al. Evaluation value of three-dimensional ultrasound quantitative indicators in the treatment of patients with decreased ovarian reserve function. Journal of Medical Imaging. 2021; 31: 1747-1750.
219. Ma X, Pei L, He S, et al. Study on the correlation between anti-Mullerian hormone level and the potential of non-quality blastocyst transplantation. China Maternal and Child Health. 2022; 37: 854-857.
220. Ma L, Qi X, Shi X. Effects of different surgical methods on anti-Mullerian hormone levels and perioperative indicators in patients with ovarian chocolate cyst. Journal of Practical Clinical Medicine. 2022; 26: 79-83.
221. Ma G, Ding F, Hu J, et al. Performance verification of anti-Millertube hormone assay kit. China Medical Device Information. 2022; 28: 12-14.
222. Lu C. Real-time shear-wave elastography combined with anti-Mullerian hormone in the diagnosis of premature ovarian failure. China Maternal and Child Health Care. 2021; 36: 1688-1690.
223. Liu X, Li G, Yang H, et al. Correlation analysis of serum AMH, FSH, InhB and sperm parameters. Laboratory Medicine. 2021; 36: 1047-1049.
224. Liu S, Zheng H. The value of anti-Mullerian hormone combined with sex hormone in the diagnosis of polycystic ovary syndrome. Journal of Practical Gynecology Endocrinology (Electronic) 2021; 8: 69-71.
225. Liu H, Zhu D, Li Y, et al. Effect of laparoscopic versus hysteroscopic surgery on local cervical microcirculation and serum AMH in the treatment of type Ⅱ submucosal uterine fibroids. Chinese Journal of Family Planning. 2022; 30: 411-415.
226. Liu H. Clinical value of anti-Mullerian hormone combined with sex hormone detection in the diagnosis of polycystic ovary syndrome. Current Journal of Clinical Medicine. 2022; 35: 24-25.
227. Lin Q, Ou D, Chen J. Application of serum AMH, CA125 and PRL in polycystic ovary syndrome. Chinese Health Standard Management. 2022; 13: 69-72.
228. Lin C, Zhu Y, Wang H. Application value of serum anti-Mullerian hormone in the diagnosis of polycystic ovary syndrome. Chinese Journal of Health Laboratory Science. 2022; 32: 2027-2031.
229. Liao Y, Zhao J. To evaluate the value of serum AMH detection in predicting ovarian response in polycystic ovary syndrome. Chinese Health Standard Management. 2022; 13: 94-97.
230. Liao H, Yang X. Anti-Mullerian hormone in predicting ovarian responsiveness after ovulation induction therapy in polycystic ovary syndrome. Chinese Journal of Family Planning. 2022; 30: 126-128+134.
231. Liang J, Xu G, Sun S, et al. Serum anti-Mullerian hormone levels in patients with polycystic ovary syndrome and its predictive value. China Maternal and Child Health Care. 2021; 36: 5000-5003.
232. Li Y, Sun X, Wang J. Study on the value of AMH, EMAb and tPA combined detection in prognosis assessment of patients with endometriosis. PLA Medical Journal. 2021; 33: 93-96
233. Li X, Lin X, Zheng X, et al. Effect of dehydroepiandrosterone on pregnancy outcome and follicular fluid in patients with low ovarian response. Chinese Health Standard Management. 2022; 13: 113-116.
234. Li X. The value of anti-Mullerian hormone in the diagnosis of polycystic ovary syndrome and its correlation with female sex hormone levels. Master Jilin University Changchun. 2022.
235. Li S, Li R. Predictive significance of anti-Millerian hormone on maternal and infant outcomes. Chinese Journal of Practical Gynecology and Obstetrics. 2022; 38: 958-960.
236. Li S, Tan S, Chen C, et al. Application value of anti-Mullerian hormone follicle-stimulating hormone estradiol in diagnosis of premature ovarian failure. Forum on Primary Medicine. 2022; 26: 70-72.
237. Li Q, Wang J. Carbohydrate antigen. 199 human chorionic gonadotropin β and anti-Mullerian hormone in predicting ovarian cancer in endometriosis. China Maternal and Child Health. 2022; 37: 1979-1981.
238. Li J. Effect of laparoscopic ovarian cyst removal on sex hormone and serum AMH and INHB levels in patients. Henan Journal of Surgery. 2022; 28: 176-178.
239. Li J, Wang Y, Chi H. Fertility assessment in patients with inflammatory bowel disease. Chinese Journal of Inflammatory Bowel Disease. 2021; 05: 284-288.
240. Li H, Zhu X, Liu D, et al. Effect of high serum anti-Mullerian hormone level on hyperovulatory response in patients with polycystic ovary syndrome. China Maternal and Child Health. 2022; 37: 2514-2517.
241. Li H, Wu S, Wang Y, et al. Evaluation of ovarian function during ovarian suppression therapy in premenopausal breast cancer patients: a cohort study. Chinese Journal of Reproduction and Contraception. 2022; 42: 156-164.
242. Li H, Wang M, Zhao X, et al. Clinical value of HLA-DR combined with AMH in evaluating ovarian function in young infertile women. Family Planning and Obstetrics and Gynecology of China. 2022; 14: 88-91+112.
243. Li H, Li H, Wang W. Serum microrNA-125B expression in patients with premature ovarian failure and its correlation with TCM syndromes. Anhui Pharmaceutical. 2022; 26: 509-513.
244. Li F, Wu H, Jiang Q, et al. Effect of HMG combined with letrozole on basic AMH levels of sex hormones and endometrial receptivity in patients with polycystic ovary syndrome. Journal of Clinical Psychosomatic Diseases. 2022; 28: 38-42.
245. Lei C, Ni K, Wu L, et al. Preliminary study on the relationship between anti-millerian hormone and related clinical factors. Chinese Journal of Nuclear Medicine and Molecular Imaging. 2022; 42: 221-225.
246. Jian F, Chang Y. Significance of serum AMH, FSH, LH and E_2 after operation of ovarian chocolate cyst. Zhejiang Trauma Surgery. 2021; 26: 835-836.
247. Jia J, Li X, Geng P. Serum anti-Mullerian hormone and sex hormone levels and adverse outcomes in women with polycystic ovary syndrome in the first trimester. Chinese Journal of Family Planning. 2022; 30: 1926-1928.
248. Zhang K, Ma F, Du D, et al. Establishment of AMH reference range and correlation between AMH and cardiovascular risk factors in healthy women of reproductive age in Urumqi. Chinese General Practice. 2022; 25: 2740-2745.
